# Correction: An activator of G protein-coupled receptor and MEK1/2-ERK1/2 signaling inhibits HIV-1 replication by altering viral RNA processing

**DOI:** 10.1371/journal.ppat.1012155

**Published:** 2024-04-09

**Authors:** Raymond W. Wong, Ahalya Balachandran, Peter K. Cheung, Ran Cheng, Qun Pan, Peter Stoilov, P. Richard Harrigan, Benjamin J. Blencowe, Donald R. Branch, Alan Cochrane

There is an error in the underlying blot data in part H of S2 Fig, which supports the western blot panels in part A of S11 Fig. The anti-P-p38 and Anti-p38 panels of the originally published S2H Fig are from a replicate experiment. With this notice the authors provide a corrected S2H Fig including the full-length blots that correspond to the anti-P-p38 and anti-p38 panels in Fig S11A.

The authors provide the following additional clarifications:

In the caption of Fig 1 of [1], it is stated that the lanes in Figs D-E were cropped/assembled from the same blots and the underlying blot images are available as Figs S2A and S2B of the original article [1]; however, the reassembly of these lanes was not clearly marked on the figure. To ensure best practice, the authors provide an updated version of Fig 1 indicating where lanes within the blots provided in the Supporting Information have been reassembled.

Some control blot and microscopy data were previously published in [2] and [3], but the intentional reuse of these data and attribution to these earlier publications were not reported in [1]. Specifically, DMSO-treated cells in this study were tested simultaneously with a number of other RNA processing inhibitors reported in [2] and [3]. The same representative blot and microscopy images were used in [1] as follows:

- In Fig 1E of [1], the -DMSO and +DMSO panels for lanes for all panels are the same as the -DMSO and +DMSO lanes found in Fig 4D of [2].
- In Fig 2D of [1] the DMSO + DOX panels are the same as the DMSO + DOX panels in Fig 6 of [3].

The authors apologize for the error in the published article.

**Fig 1:**
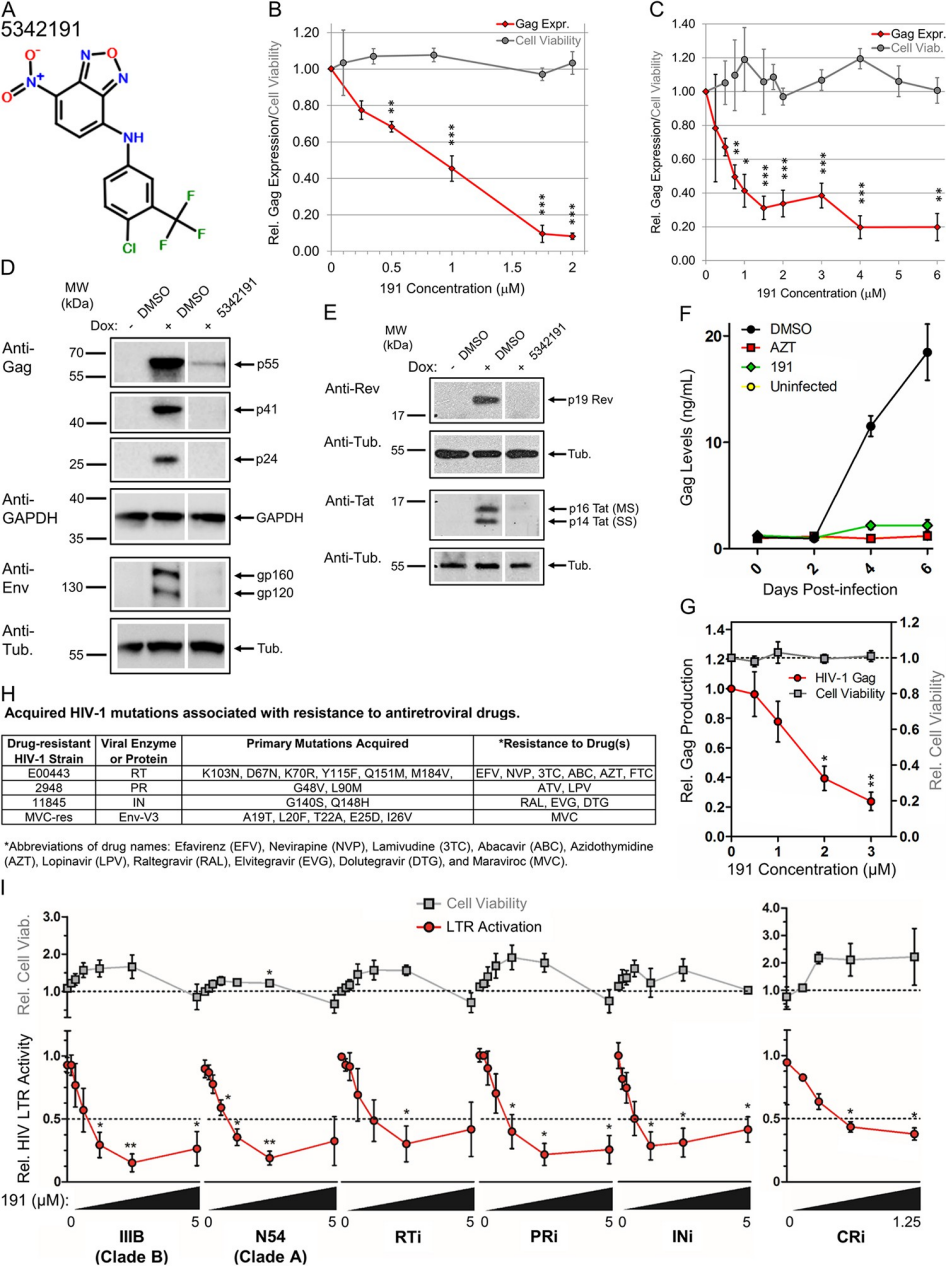
5342191 inhibits HIV-1 gene expression and replication. **(A)** 5342191 chemical structure. HeLa rtTA-HIV-Δ*Mls* (**B** and **D-E**) or CD4^+^ 24ST1NLESG T cells (**C**) were treated with indicated concentrations or IC_90_ (2 μM) of 5342191 (191), or DMSO (control) only for 4 h prior to Dox or PMA (+) induction (resp.) of HIV-1 expression for 20 h. Each treatment contained equal concentrations of DMSO solvent. After ~24 h, cell supernatants were harvested for **(B-C)** p24^CA^ ELISA of HIV-1 Gag expression (black diamonds) and XTT assay of cell viability (gray circles; n ≥ 4–5, mean, s.e.m.) while (**D-E**) cell lysates (~30 μg) were analyzed by immunoblot for expression of (**D**) HIV-1 structural proteins: Gag (p55, p41, and p24) and Env (gp160 and gp120), (**E**) viral regulatory factors: Rev (p19) and Tat (p16 and p14), and internal loading controls: GAPDH or α-tubulin (n ≥ 4–6, mean, s.e.m.). Position of molecular weight (MW) standards were marked adjacent to each gel/blot. Lanes in (**D-E,** resp.) were cropped/assembled from the same blots (S2A and S2B Fig). (**F**-**G**) Primary CD4^+^ T cells (PBMCs) were infected or left uninfected (yellow circles) with HIV-1 *Ba-L*, treated with 3 μM 5342191 (191, green diamonds), 3.7 μM AZT (red boxes), or DMSO only (black circles), which was partially replenished with fresh drug & media after 4 days, and cell supernatant harvested every 2 days for p24^CA^ ELISA to monitor effects on (**F**) HIV-1 growth over 0–6 days and (**G**) dose-response of 0–3 μM of 5342191 on HIV-1 replication (red circles) and cell viability (gray boxes, by trypan blue exclusion) on day 6. Data from (**F**-**G**) are n = 4 from 1 donor, representative of 4 different donors, mean, s.e.m. (**H**-**I**) CEM-GXR cells were infected with WT (*IIIB* or *N54*) or RT inhibitor (i), PRi, INi, or CRi-resistant strains of HIV-1 described in **(H)**, treated with 0, 0.15, 0.3, 0.6, 1.25, 2.5, or 5 μM of 5342191 (gradient bar, except CRi: 0–1.25 μM), and their effects on **(I)** HIV-1 LTR activation (red circles) and cell viability (gray boxes) quantified from GFP fluorescence and live-cell counts, respectively, by flow cytometry after 3 days of culture (n ≥ 3, except CRi: n ≥ 2–3, mean, s.e.m.). Dotted-black lines in (**G** and **I**) mark 100% cell viability or IC_50_. All results are relative and statistically compared to treatment with 0 μM of compound. In (**E**), the DMSO +/- Dox lanes of the blot from [2] were reused since compound 5342191 was tested in parallel with other RNA processing inhibitors (digoxin) and were the best representative lanes of treatment controls.

**Fig 2:**
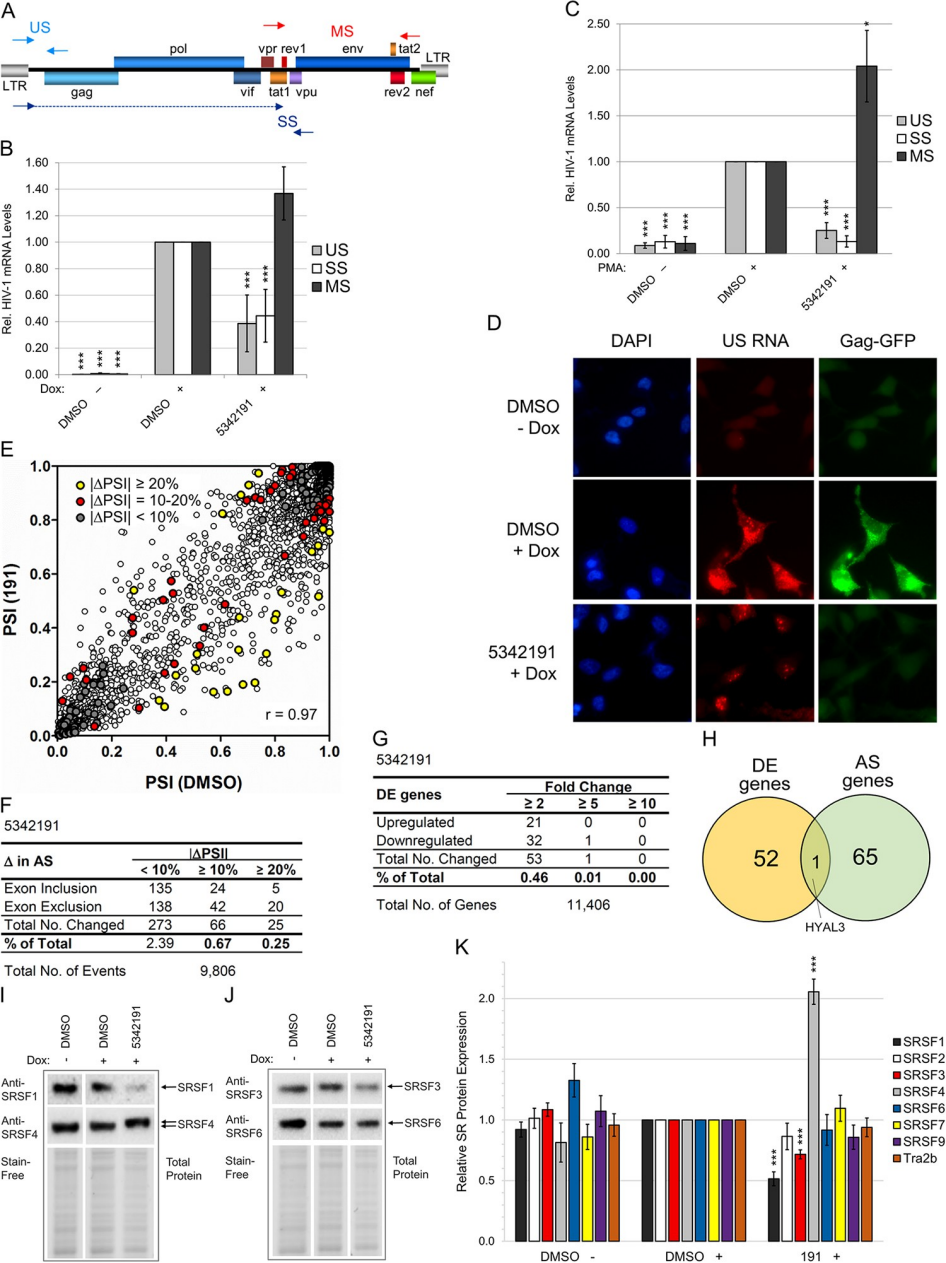
5342191 affects HIV-1 RNA processing and expression/modification of SR splicing factors with limited perturbation of AS and expression of host RNAs. HeLa rtTA-HIV-Δ*Mls* (**A**-**B** and **E**-**H**), 24ST1NLESG T (**C**), and HeLa rtTA-HIV(Gag-GFP) cells (**D**) and (**I**-**K**) were treated with/without IC_80_-IC_90_ of 5342191 (2, 4, 2, and 2.5 μM, resp.) and Dox per Fig 1 and assayed as follows. (**A**-**C**) Quantitation of the relative expression of HIV-1 US (gray), SS (white), and MS RNAs (black) in cells by qRT-PCR. (**A**) Diagram of the HIV-1 genome indicating position of primers used in amplification. Solid arrow heads denote start while dashed-lined arrows represent exon coverage. (**B**-**C**) Graph of RNA levels quantified from HeLa rtTA-HIV-Δ*Mls* and 24ST1NLESG T cells (resp.) treated with/without 5342191 (n ≥ 3, mean, s.e.m.). Results are relative and statistically compared to DMSO (+) for each RNA class. (**D**) Trafficking of US RNAs (labeled with Texas Red) detected by FISH (representative of n ≥ 3). Nuclei were detected by DAPI stain, Gag-GFP expression by GFP, and images captured at 630x magnification. (**E**-**H**) RNA-Seq quantifying the AS of host RNAs (mean PSI) from 9,806 exon inclusion/exclusion events examined and DE genes [mean fold change (Δ)] from 11,406 host RNAs detected from 5342191 or DMSO-treated cells (S1 and S3 Tables; n = 2, mean). (**E**) Scatterplot of PSIs displaying differences in AS between 5342191 (y-axis) and DMSO (x-axis) with significant ΔPSIs (p <0.05) indicated by colored circles as follows: <10% (gray), 10–20% (yellow), and ≥ 20% (red). (**F**) Total number and percentage (%) of AS events altered (ΔPSI ≥ 10% and 20%), (**G**) total number and % of DE genes changed (≥ 2, 5, and 10 fold), and (**H**) Venn diagram of the AS (≥ 10%) and DE genes (≥ 2 fold) affected in common. (**I, J,** and S5 Fig) Representative immunoblots and (**K**) graph quantifying the accumulation (and modification) of endogenous SR proteins from lysates of treated cells (~30 μg, n ≥ 3, mean, s.e.m.). Results are relative and statistically compared to DMSO (+). β-actin (**B**-**C**) and Stain-Free-labeled total proteins (**I**-**K**) served as internal loading controls for normalization of RNA and protein data, respectively. In (**D**), the image for US RNA in DMSO + Dox treated cells was reused in [3] since these compounds were performed in parallel with 5342191 and the best available representative image of treatment controls.

## Supporting information

S2 Fig Gel/blots used for representative figures. Lanes from continuous and unexcised gel/blots were cropped and rearranged for Fig 1D (**A**) and 1E (**B**), Fig 2I and 2J (**C**-**D**), S5 Fig (**E**), S6 Fig (**F**), S7A Fig (**G**), S11A Fig (**H**), S11B (**I**), S11C Fig (**J**), S11D Fig (**K**-**L**), and S13C Fig (**M**). The experimental conditions used in each gel/blot(s) can be found under the representative Fig(s) or Supporting Fig(s) listed and associated with it.
(TIF)
