# Supplementary figures and images for: An activator of G protein-coupled receptor and MEK1/2-ERK1/2 signaling inhibits HIV-1 replication by altering viral RNA processing
Source: PLoS Pathog. 2020 Feb 18;16(2):e1008307. doi: 10.1371/journal.ppat.1008307 (PMC7048317; doi:10.1371/journal.ppat.1008307)

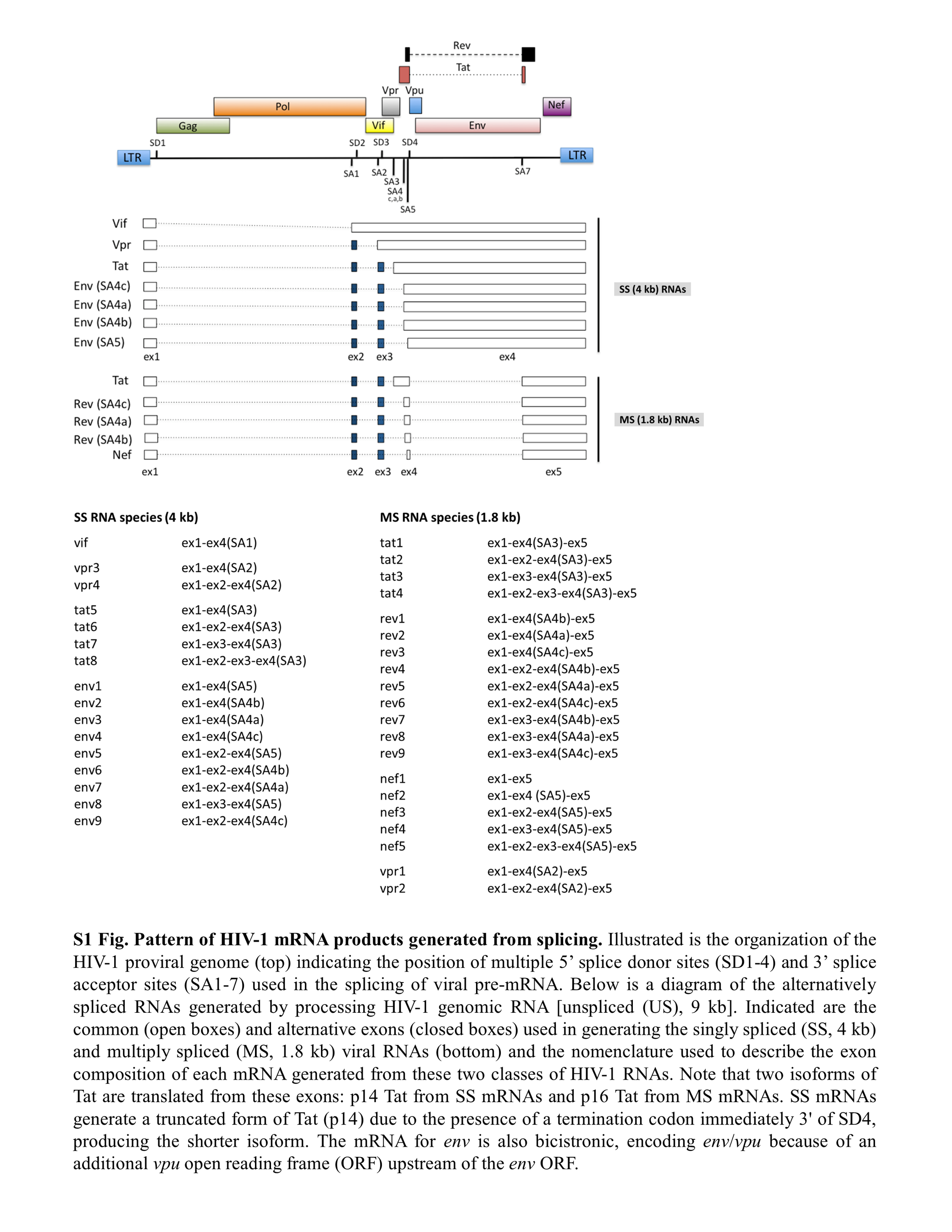

Supplement: S1 Fig — Illustrated is the organization of the HIV-1 proviral genome (top) indicating the position of multiple 5’ splice donor sites (SD1-4) and 3’ splice acceptor sites (SA1-7) used in the splicing of viral pre-mRNA. Below is a diagram of the alternatively spliced RNAs generated by processing HIV-1 genomic RNA [unspliced (US), 9 kb]. Indicated are the common (open boxes) and alternative exons (closed boxes) used in generating the singly spliced (SS, 4 kb) and multiply spliced (MS, 1.8 kb) viral RNAs (bottom) and the nomenclature used to describe the exon composition of each mRNA generated from these two classes of HIV-1 RNAs. Note that two isoforms of Tat are translated from these exons: p14 Tat from SS mRNAs and p16 Tat from MS mRNAs. SS mRNAs generate a truncated form of Tat (p14) due to the presence of a termination codon immediately 3' of SD4, producing the shorter isoform. The mRNA for env is also bicistronic, encoding env/vpu because of an additional vpu open reading frame (ORF) upstream of the env ORF. (TIF) [file ppat.1008307.s007.tif]

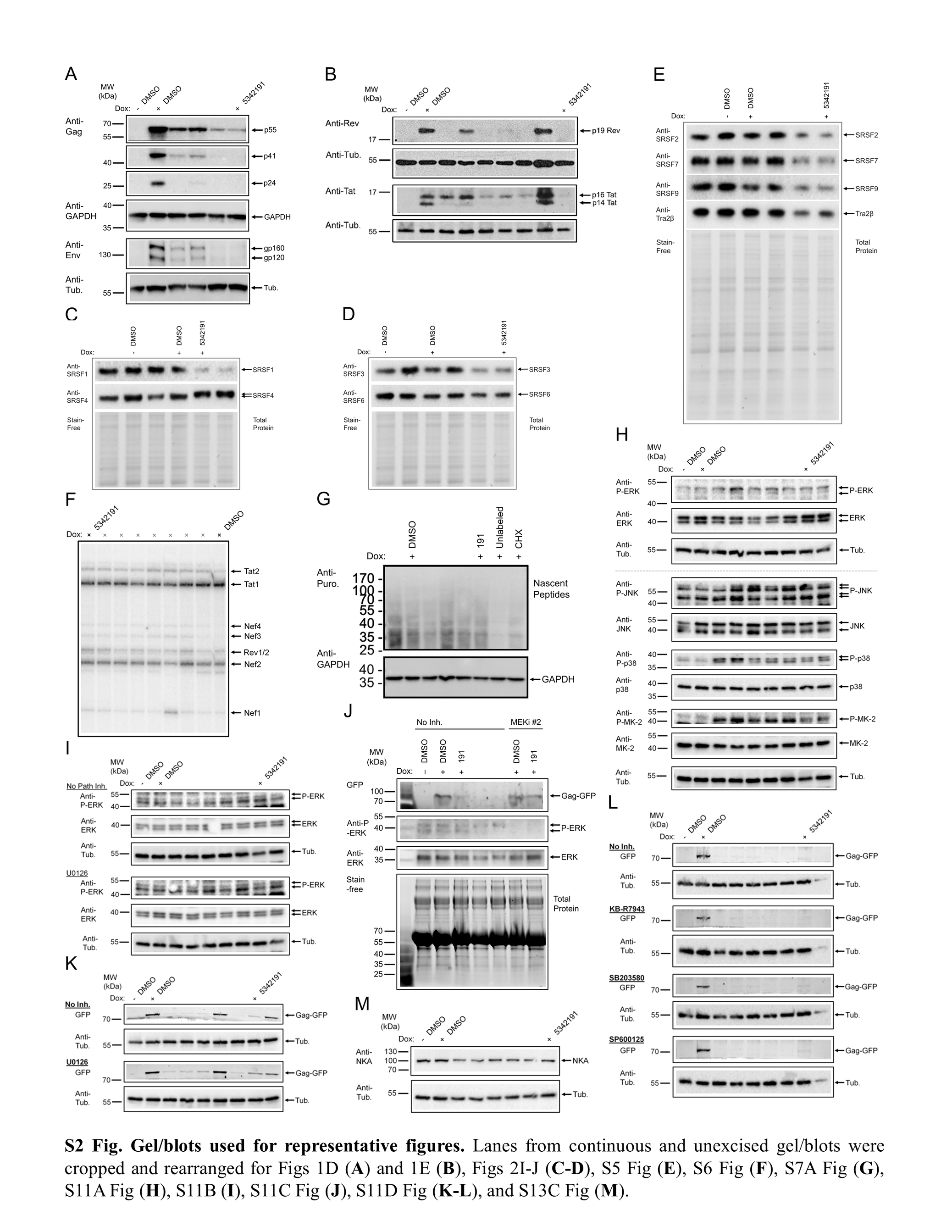

Supplement: S2 Fig — Lanes from continuous and unexcised gel/blots were cropped and rearranged for Fig 1D (A) and 1E (B), Fig 2I and 2J (C-D), S5 Fig (E), S6 Fig (F), S7A Fig (G), S11A Fig (H), S11B (I), S11C Fig (J), S11D Fig (K-L), and S13C Fig (M). (TIF) [file ppat.1008307.s008.tif]

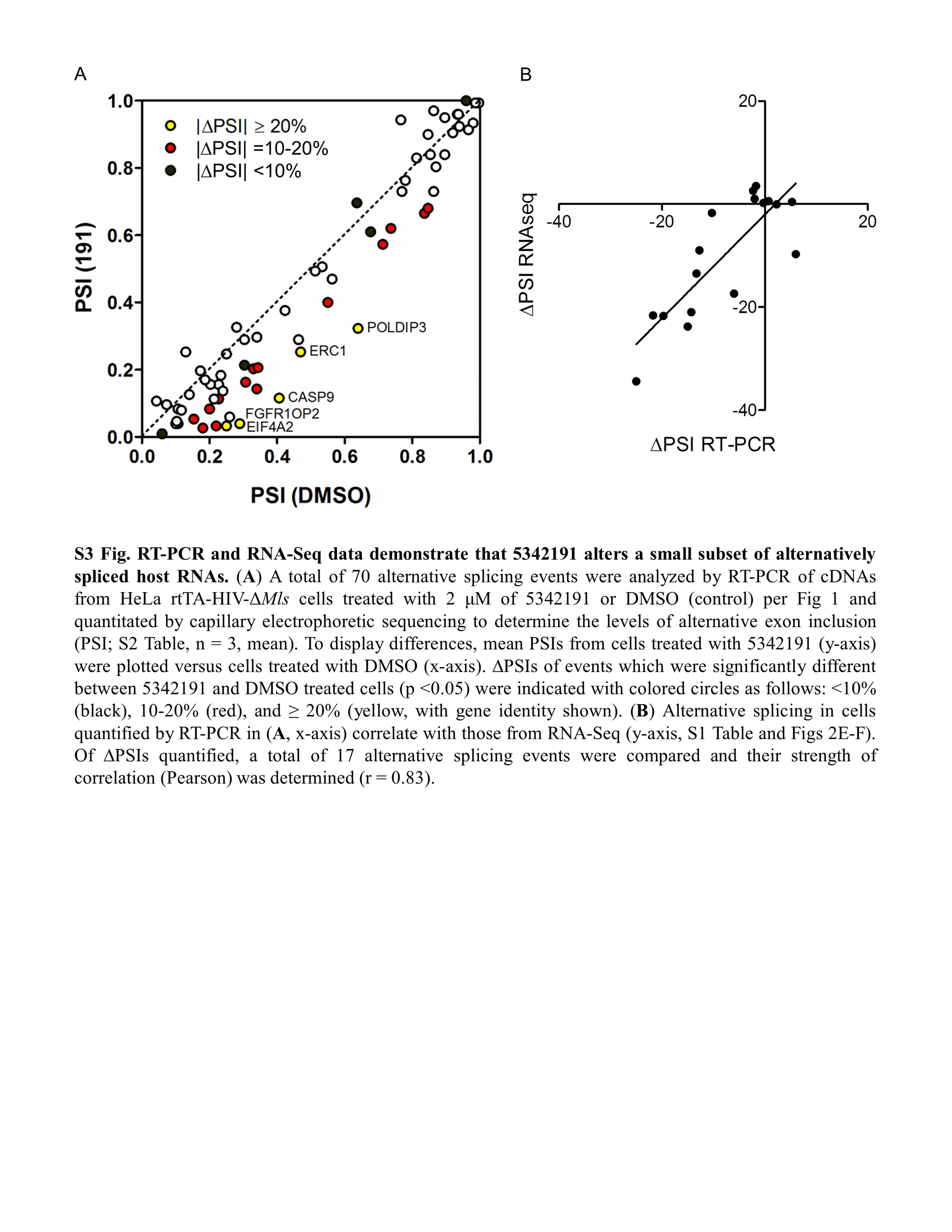

Supplement: S3 Fig — (A) A total of 70 alternative splicing events were analyzed by RT-PCR of cDNAs from HeLa rtTA-HIV-ΔMls cells treated with 2 μM of 5342191 or DMSO (control) per Fig 1 and quantitated by capillary electrophoretic sequencing to determine the levels of alternative exon inclusion (PSI; S2 Table, n = 3, mean). To display differences, mean PSIs from cells treated with 5342191 (y-axis) were plotted versus cells treated with DMSO (x-axis). ΔPSIs of events which were significantly different between 5342191 and DMSO treated cells (p <0.05) were indicated with colored circles as follows: <10% (black), 10–20% (red), and ≥ 20% (yellow, with gene identity shown). (B) Alternative splicing in cells quantified by RT-PCR in (A, x-axis) correlate with those from RNA-Seq (y-axis, S1 Table and Fig 2E and 2F). Of ΔPSIs quantified, a total of 17 alternative splicing events were compared and their strength of correlation (Pearson) was determined (r = 0.83). (TIF) [file ppat.1008307.s009.tif]

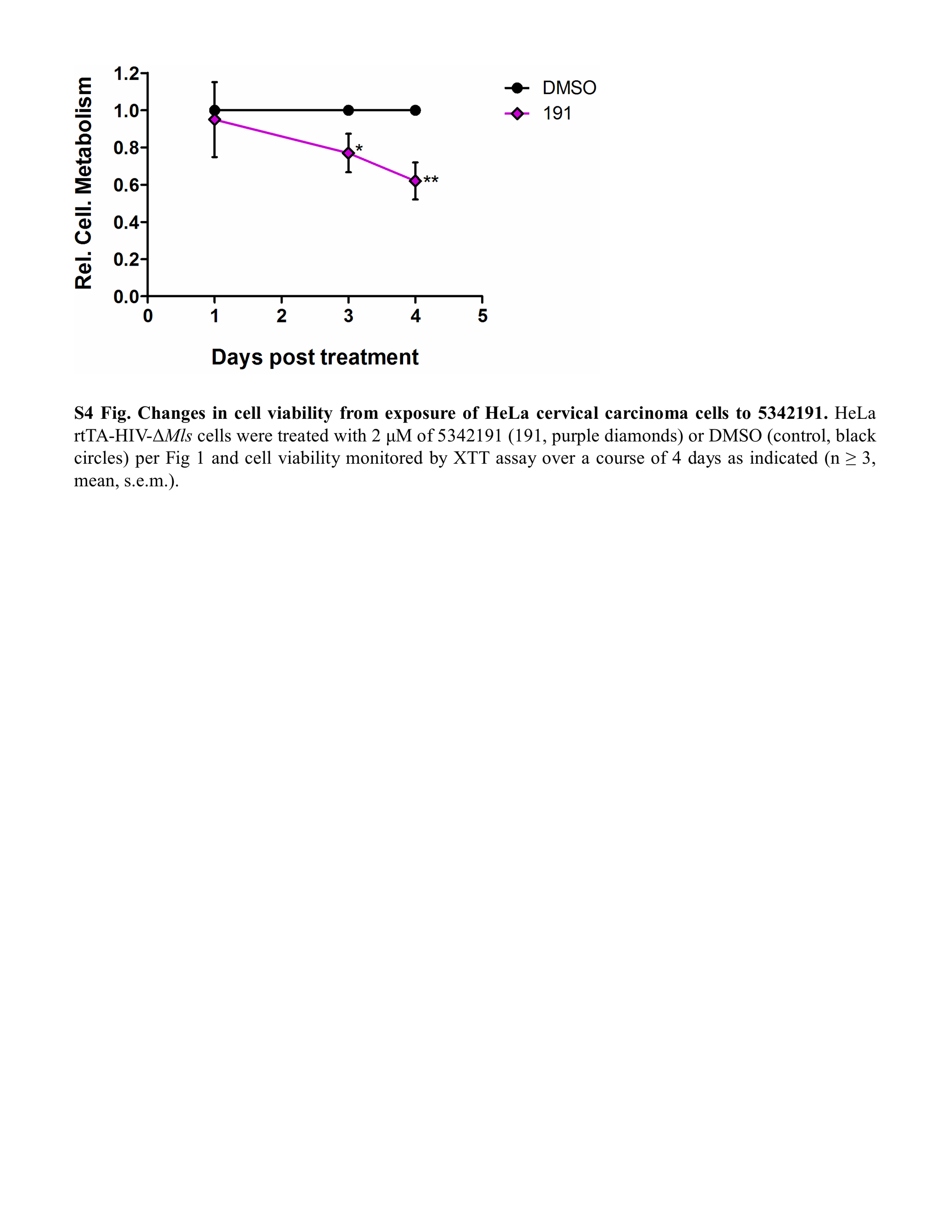

Supplement: S4 Fig — HeLa rtTA-HIV-ΔMls cells were treated with 2 μM of 5342191 (191, purple diamonds) or DMSO (control, black circles) per Fig 1 and cell viability monitored by XTT assay over a course of 4 days as indicated (n ≥ 3, mean, s.e.m.). (TIF) [file ppat.1008307.s010.tif]

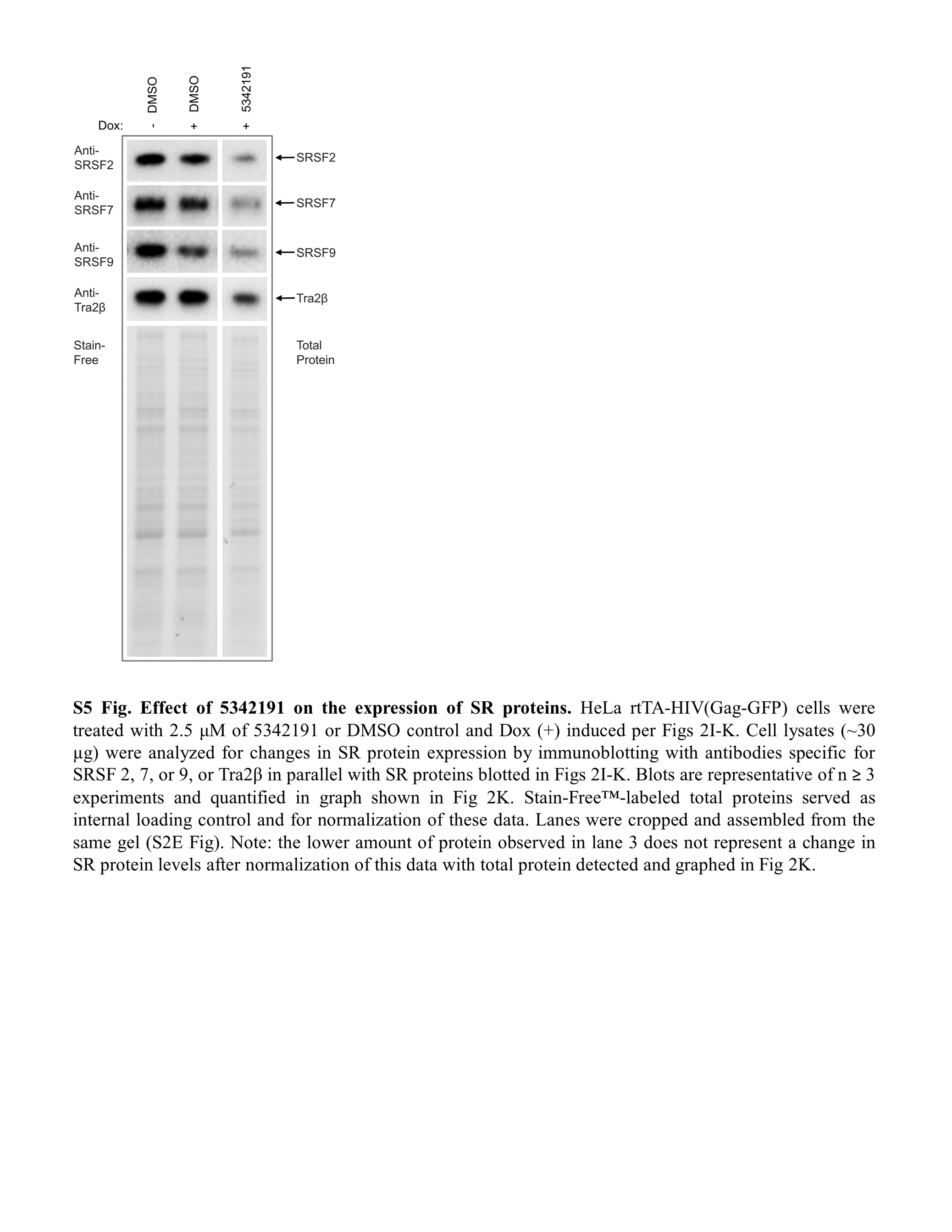

Supplement: S5 Fig — HeLa rtTA-HIV(Gag-GFP) cells were treated with 2.5 μM of 5342191 or DMSO control and Dox (+) induced per Fig 2I–2K. Cell lysates (~30 μg) were analyzed for changes in SR protein expression by immunoblotting with antibodies specific for SRSF 2, 7, or 9, or Tra2β in parallel with SR proteins blotted in Fig 2I–2K. Blots are representative of n ≥ 3 experiments and quantified in graph shown in Fig 2K. Stain-Free-labeled total proteins served as internal loading control and for normalization of these data. Lanes were cropped and assembled from the same gel (S2E Fig). Note: the lower amount of protein observed in lane 3 does not represent a change in SR protein levels after normalization of this data with total protein detected and graphed in Fig 2K. (TIF) [file ppat.1008307.s011.tif]

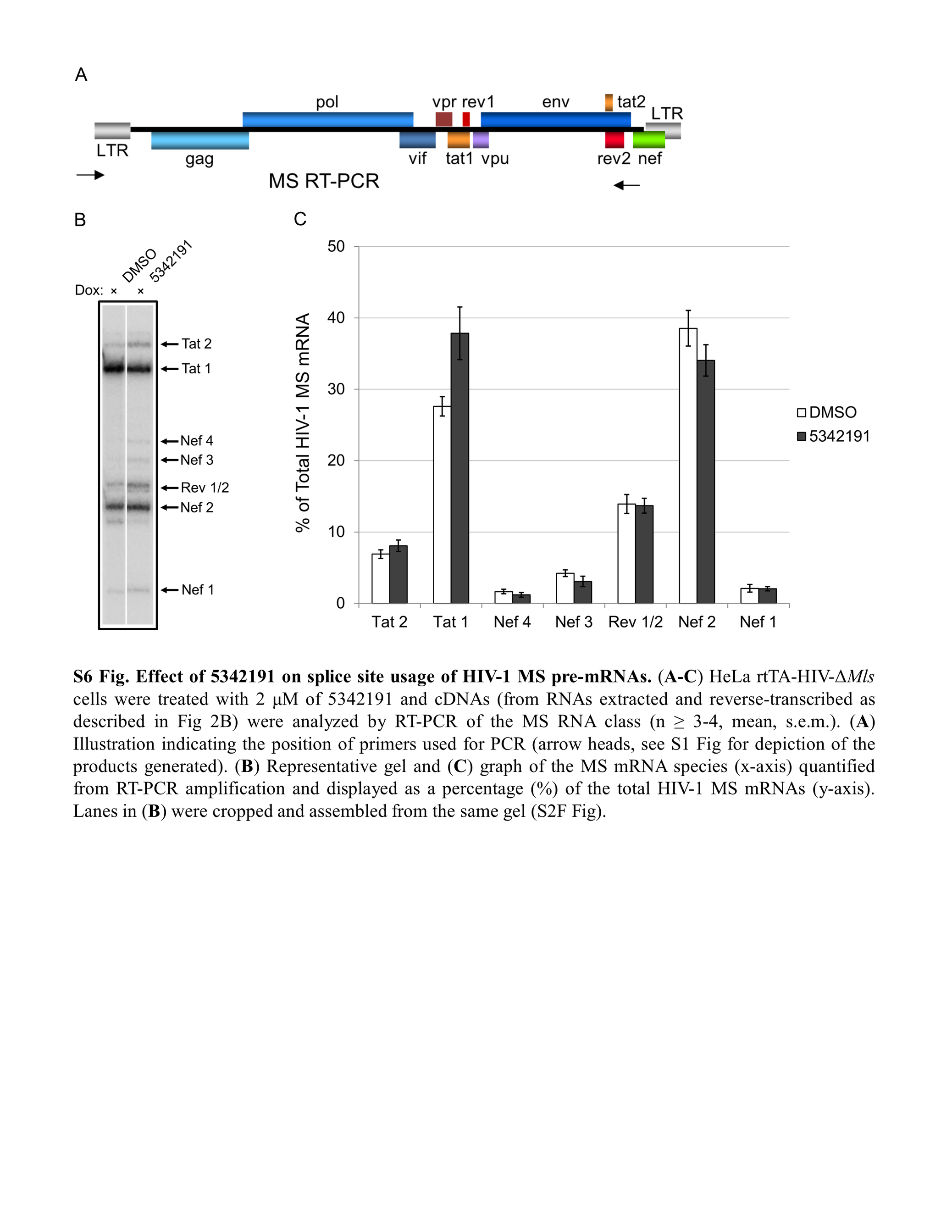

Supplement: S6 Fig — (A-C) HeLa rtTA-HIV-ΔMls cells were treated with 2 μM of 5342191 and cDNAs (from RNAs extracted and reverse-transcribed as described in Fig 2B) were analyzed by RT-PCR of the MS RNA class (n ≥ 3–4, mean, s.e.m.). (A) Illustration indicating the position of primers used for PCR (arrow heads, see S1 Fig for depiction of the products generated). (B) Representative gel and (C) graph of the MS mRNA species (x-axis) quantified from RT-PCR amplification and displayed as a percentage (%) of the total HIV-1 MS mRNAs (y-axis). Lanes in (B) were cropped and assembled from the same gel (S2F Fig). (TIF) [file ppat.1008307.s012.tif]

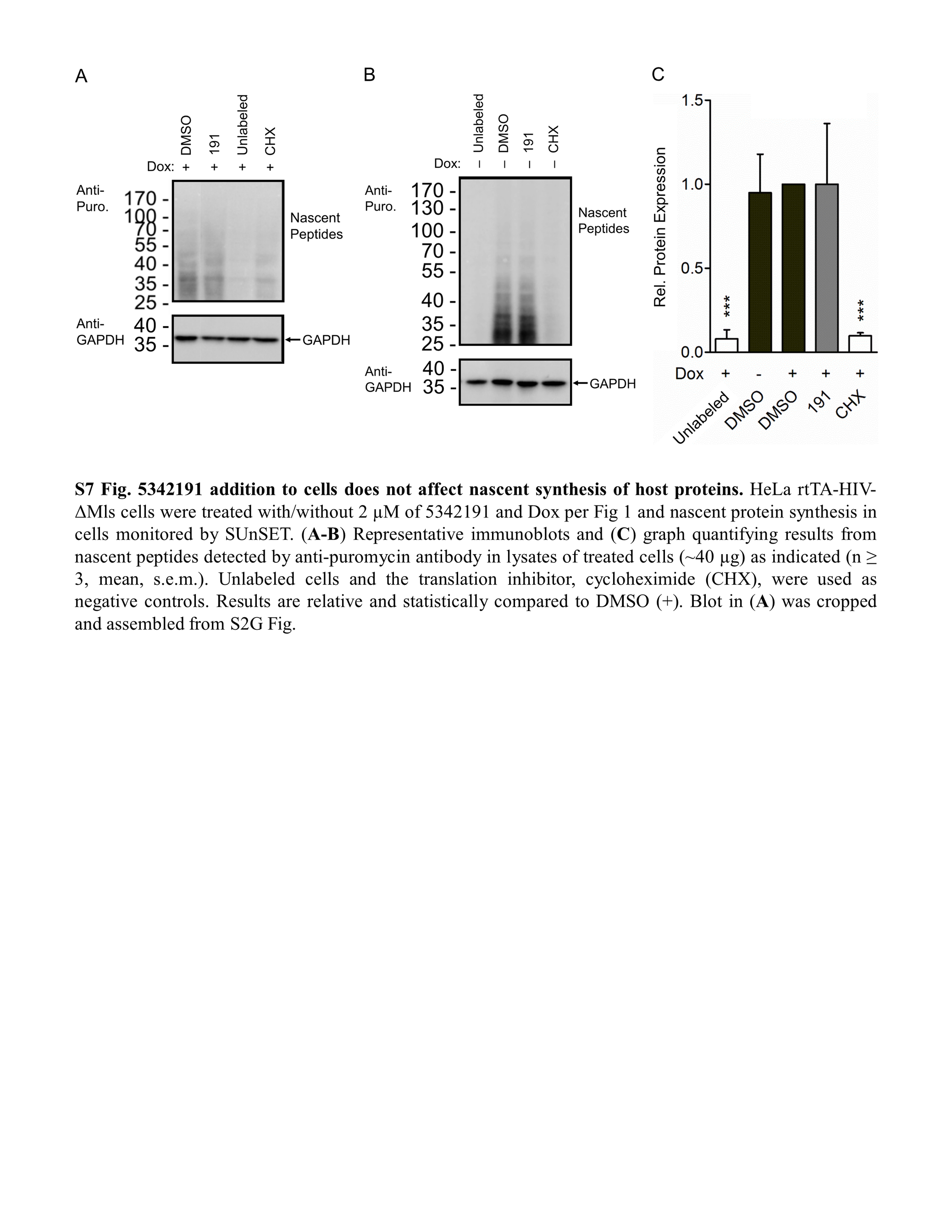

Supplement: S7 Fig — HeLa rtTA-HIV-ΔMls cells were treated with/without 2 μM of 5342191 and Dox per Fig 1 and nascent protein synthesis in cells monitored by SUnSET. (A-B) Representative immunoblots and (C) graph quantifying results from nascent peptides detected by anti-puromycin antibody in lysates of treated cells (~40 μg) as indicated (n ≥ 3, mean, s.e.m.). Unlabeled cells and the translation inhibitor, cycloheximide (CHX), were used as negative controls. Results are relative and statistically compared to DMSO (+). Blot in (A) was cropped and assembled from S2G Fig. (TIF) [file ppat.1008307.s013.tif]

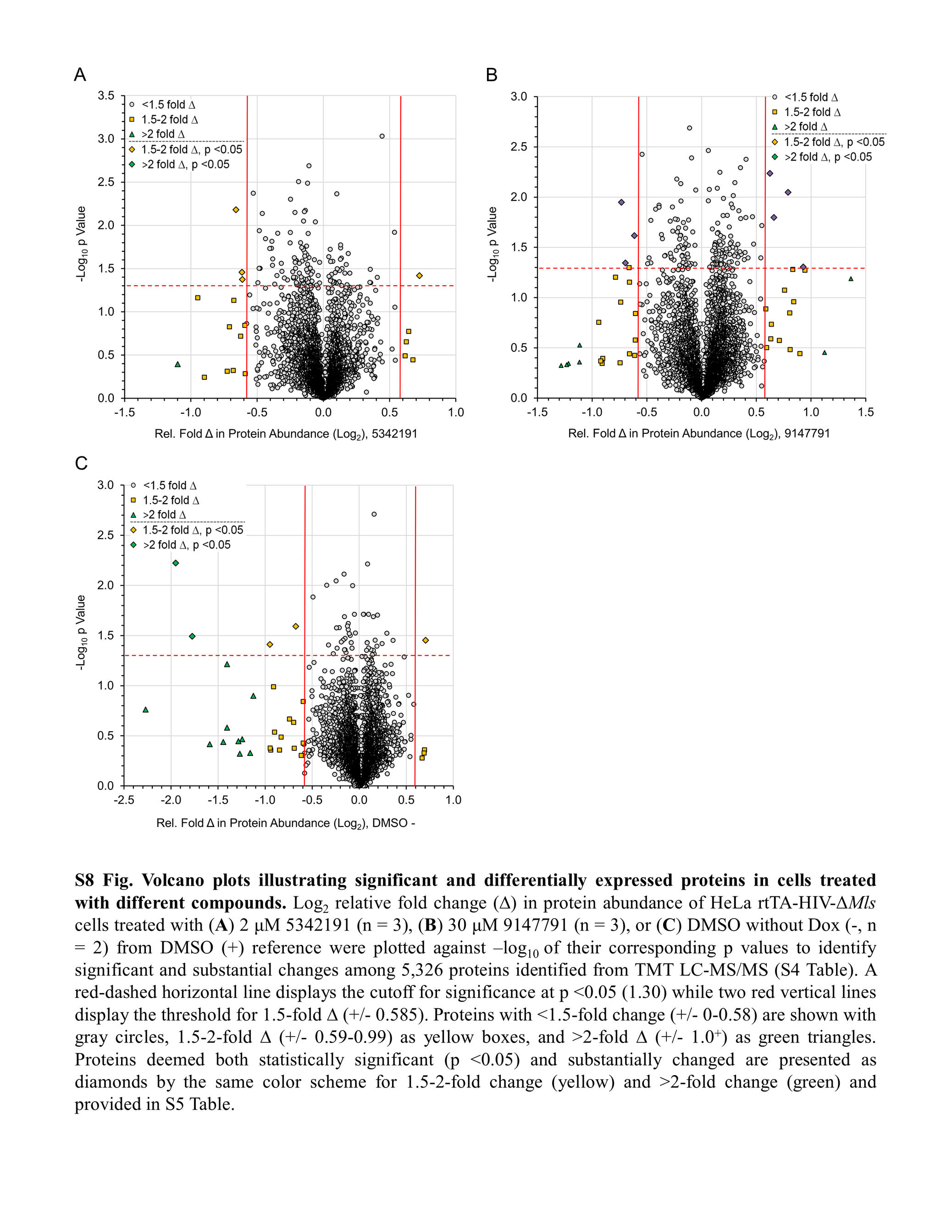

Supplement: S8 Fig — Log2 relative fold change (Δ) in protein abundance of HeLa rtTA-HIV-ΔMls cells treated with (A) 2 μM 5342191 (n = 3), (B) 30 μM 9147791 (n = 3), or (C) DMSO without Dox (-, n = 2) from DMSO (+) reference were plotted against–log10 of their corresponding p values to identify significant and substantial changes among 5,326 proteins identified from TMT LC-MS/MS (S4 Table). A red-dashed horizontal line displays the cut-off for significance at p <0.05 (1.30) while two red vertical lines display the threshold for 1.5-fold Δ (+/- 0.585). Proteins with <1.5-fold change (+/- 0–0.58) are shown with gray circles, 1.5-2-fold Δ (+/- 0.59–0.99) as yellow boxes, and >2-fold Δ (+/- 1.0+) as green triangles. Proteins deemed both statistically significant (p <0.05) and substantially changed are presented as diamonds by the same color scheme for 1.5-2-fold change (yellow) and >2-fold change (green) and provided in S5 Table. (TIF) [file ppat.1008307.s014.tif]

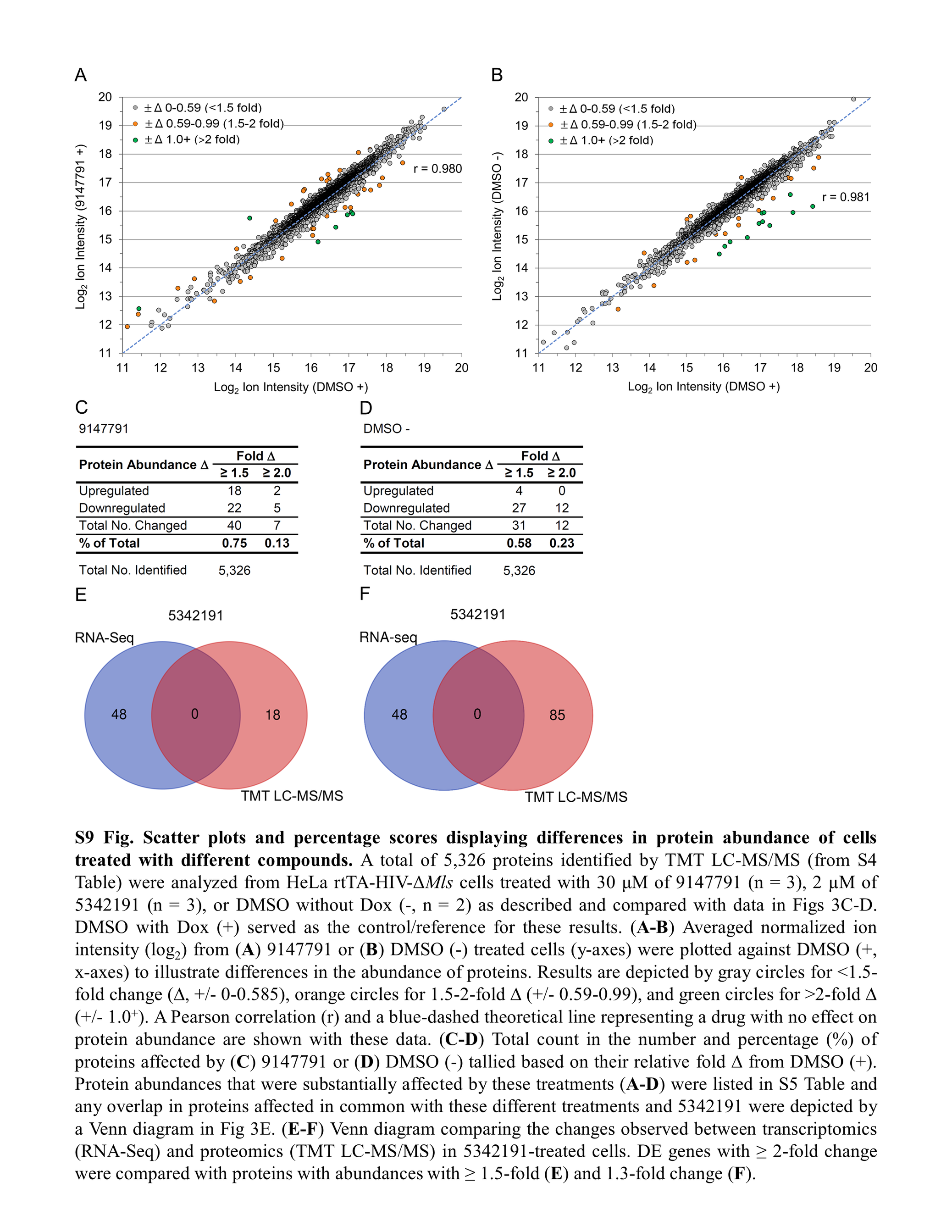

Supplement: S9 Fig — A total of 5,326 proteins identified by TMT LC-MS/MS (from S4 Table) were analyzed from HeLa rtTA-HIV-ΔMls cells treated with 30 μM of 9147791 (n = 3), 2 μM of 5342191 (n = 3), or DMSO without Dox (-, n = 2) as described and compared with data in Fig 3C and 3D. DMSO with Dox (+) served as the control/reference for these results. (A-B) Averaged normalized ion intensity (log2) from (A) 9147791 or (B) DMSO (-) treated cells (y-axes) were plotted against DMSO (+, x-axes) to illustrate differences in the abundance of proteins. Results are depicted by gray circles for <1.5-fold change (Δ, +/- 0–0.585), orange circles for 1.5-2-fold Δ (+/- 0.59–0.99), and green circles for >2-fold Δ (+/- 1.0+). A Pearson correlation (r) and a blue-dashed theoretical line representing a drug with no effect on protein abundance are shown with these data. (C-D) Total count in the number and percentage (%) of proteins affected by (C) 9147791 or (D) DMSO (-) tallied based on their relative fold Δ from DMSO (+). Protein abundances that were substantially affected by these treatments (A-D) were listed in S5 Table and any overlap in proteins affected in common with these different treatments and 5342191 were depicted by a Venn diagram in Fig 3E. (E-F) Venn diagram comparing the changes observed between transcriptomics (RNA-Seq) and proteomics (TMT LC-MS/MS) in 5342191-treated cells. DE genes with ≥ 2-fold change were compared with proteins with abundances with ≥ 1.5-fold (E) and 1.3-fold change (F). (TIF) [file ppat.1008307.s015.tif]

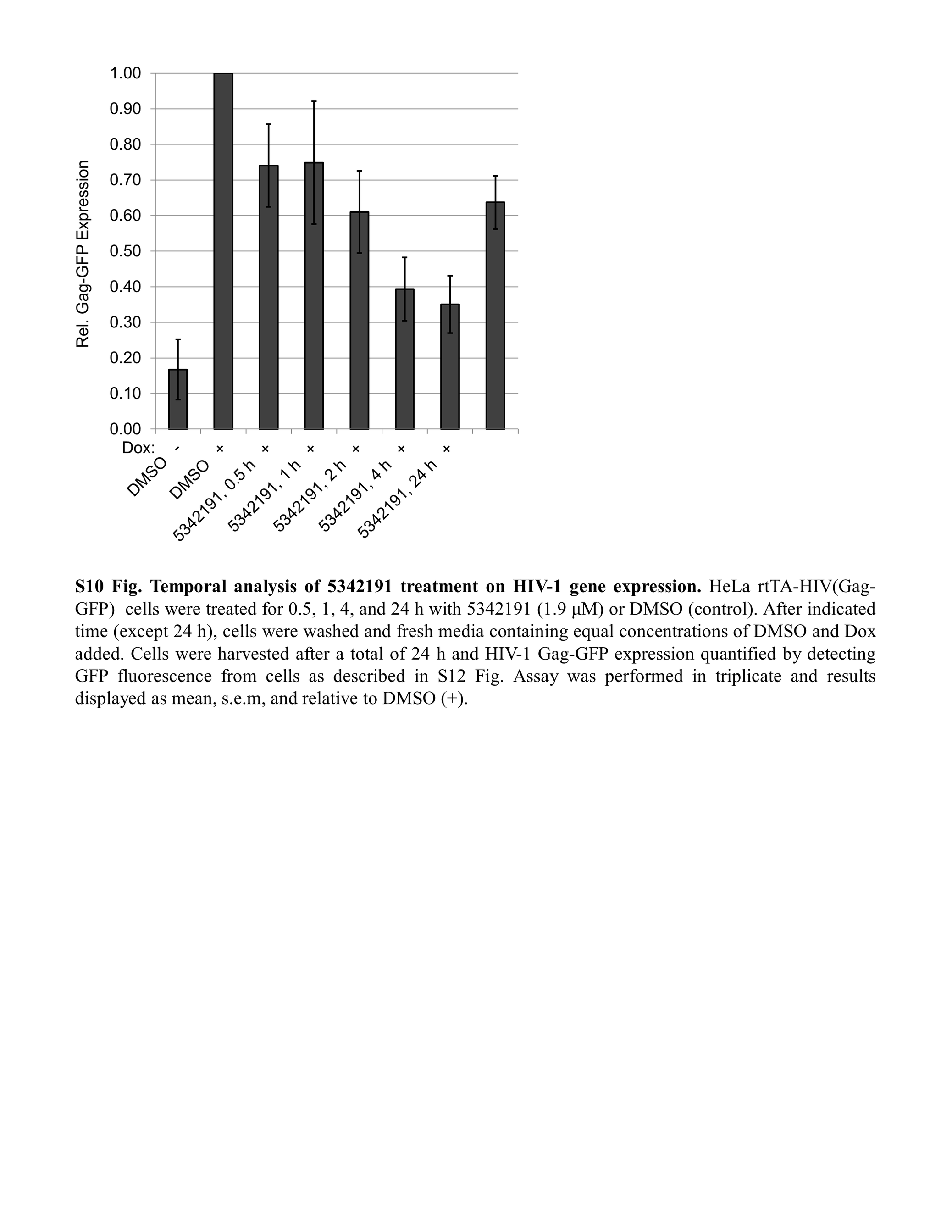

Supplement: S10 Fig — HeLa rtTA-HIV(Gag-GFP) cells were treated for 0.5, 1, 4, and 24 h with 5342191 (1.9 μM) or DMSO (control). After indicated time (except 24 h), cells were washed and fresh media containing equal concentrations of DMSO and Dox added. Cells were harvested after a total of 24 h and HIV-1 Gag-GFP expression quantified by detecting GFP fluorescence from cells as described in S12 Fig. Assay was performed in triplicate and results displayed as mean, s.e.m, and relative to DMSO (+). (TIF) [file ppat.1008307.s016.tif]

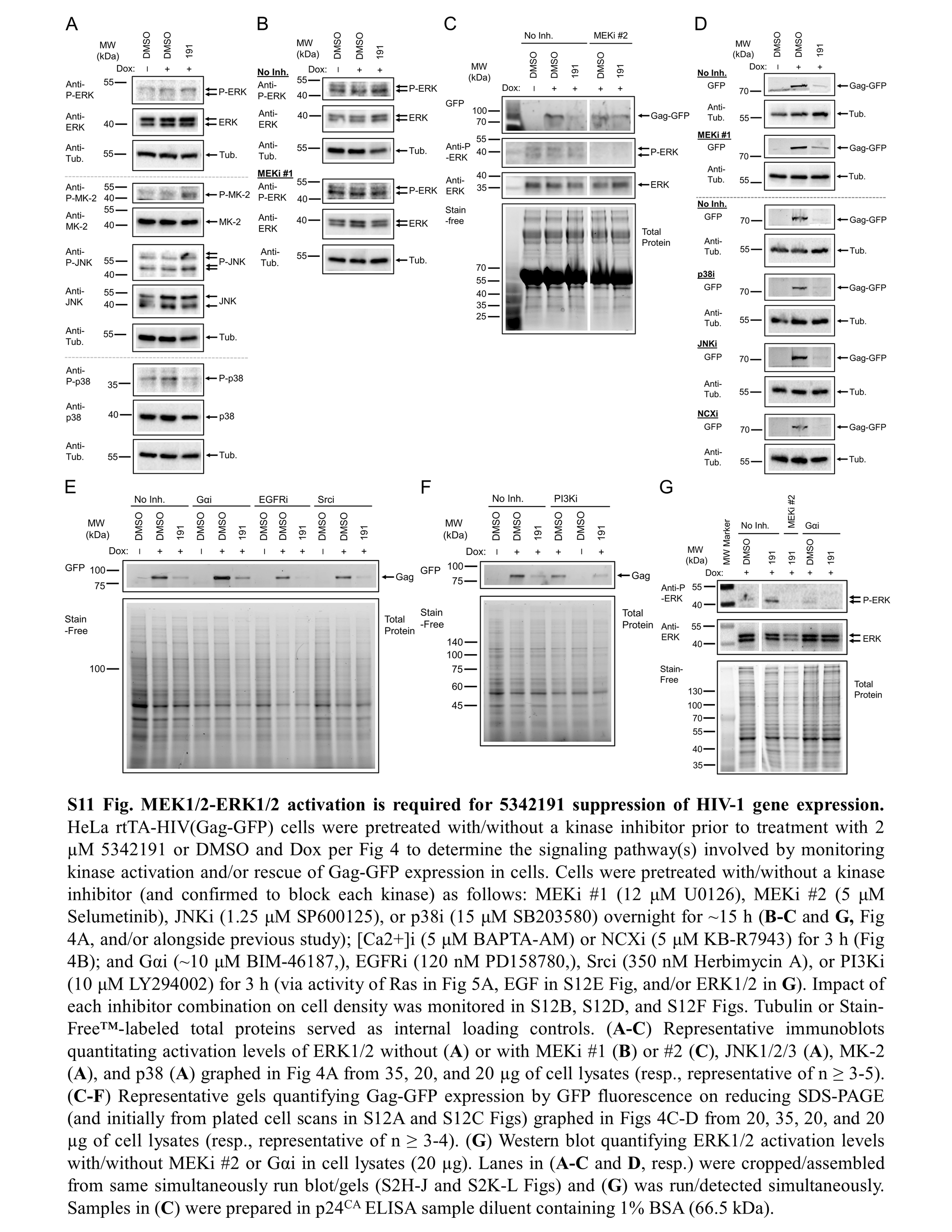

Supplement: S11 Fig — HeLa rtTA-HIV(Gag-GFP) cells were pretreated with/without a kinase inhibitor prior to treatment with 2 μM 5342191 or DMSO and Dox per Fig 4 to determine the signaling pathway(s) involved by monitoring kinase activation and/or rescue of Gag-GFP expression in cells. Cells were pretreated with/without a kinase inhibitor (and confirmed to block each kinase) as follows: MEKi #1 (12 μM U0126), MEKi #2 (5 μM Selumetinib), JNKi (1.25 μM SP600125), or p38i (15 μM SB203580) overnight for ~15 h (B-C and G, Fig 4A, and/or alongside previous study); [Ca2+]i (5 μM BAPTA-AM) or NCXi (5 μM KB-R7943) for 3 h (Fig 4B); and Gαi (~10 μM BIM-46187,), EGFRi (120 nM PD158780,), Srci (350 nM Herbimycin A), or PI3Ki (10 μM LY294002) for 3 h (via activity of Ras in Fig 5A, EGF in S12E Fig, and/or ERK1/2 in G). Impact of each inhibitor combination on cell density was monitored in S12B, S12D and S12F Fig. Tubulin or Stain-Free-labeled total proteins served as internal loading controls. (A-C) Representative immunoblots quantitating activation levels of ERK1/2 without (A) or with MEKi #1 (B) or #2 (C), JNK1/2/3 (A), MK-2 (A), and p38 (A) graphed in Fig 4A from 35, 20, and 20 μg of cell lysates (resp., representative of n ≥ 3–5). (C-F) Representative gels quantifying Gag-GFP expression by GFP fluorescence on reducing SDS-PAGE (and initially from plated cell scans in S12A and S12C Fig) graphed in Fig 4C and 4D from 20, 35, 20, and 20 μg of cell lysates (resp., representative of n ≥ 3–4). (G) Western blot quantifying ERK1/2 activation levels with/without MEKi #2 or Gαi in cell lysates (20 μg). Lanes in (A-C and D, resp.) were cropped/assembled from same simultaneously run blot/gels (S2H–S2J and S2K–S2L Fig) and (G) was run/detected simultaneously. Samples in (C) were prepared in p24CA ELISA sample diluent containing 1% BSA (66.5 kDa). (TIF) [file ppat.1008307.s017.tif]

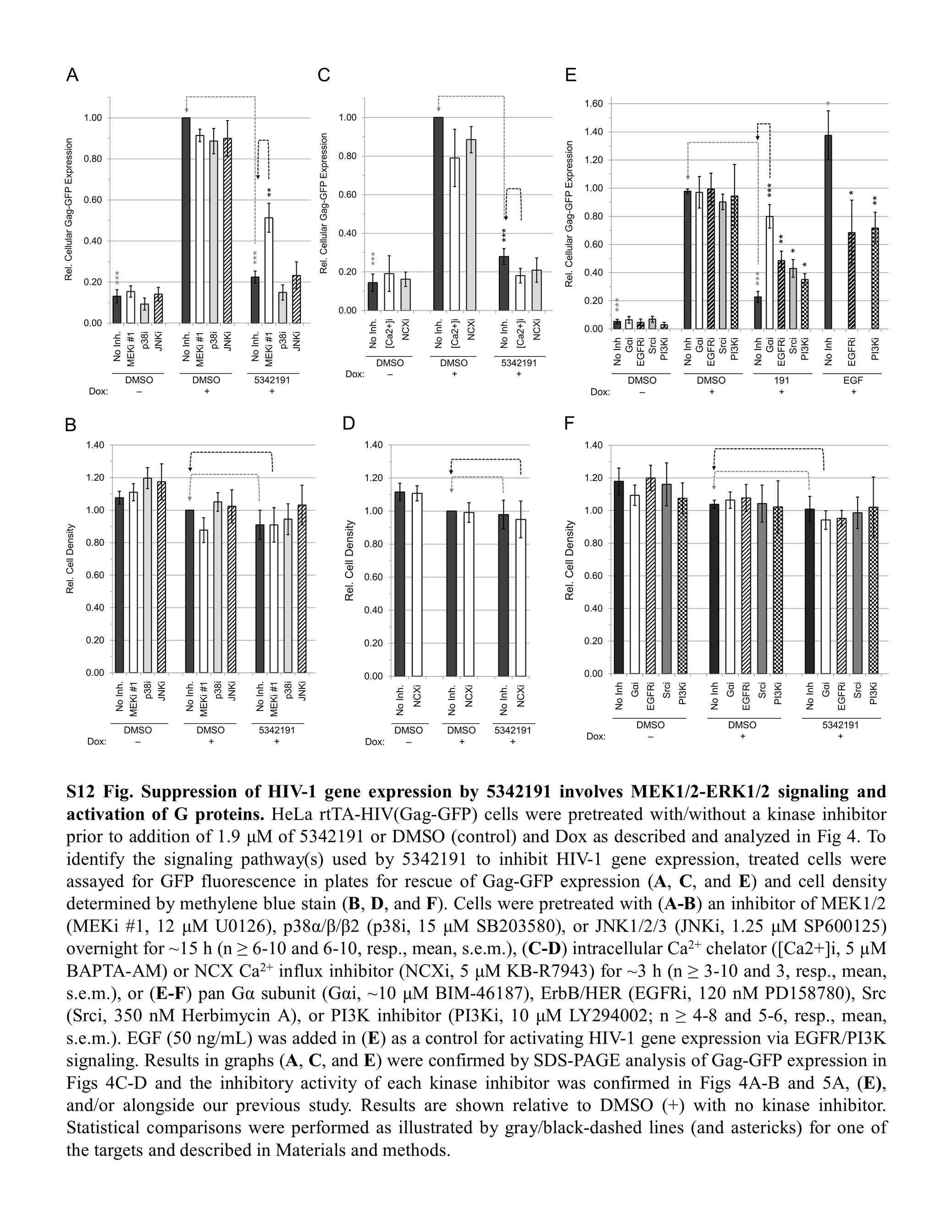

Supplement: S12 Fig — HeLa rtTA-HIV(Gag-GFP) cells were pretreated with/without a kinase inhibitor prior to addition of 1.9 μM of 5342191 or DMSO (control) and Dox as described and analyzed in Fig 4. To identify the signaling pathway(s) used by 5342191 to inhibit HIV-1 gene expression, treated cells were assayed for GFP fluorescence in plates for rescue of Gag-GFP expression (A, C, and E) and cell density determined by methylene blue stain (B, D, and F). Cells were pretreated with (A-B) an inhibitor of MEK1/2 (MEKi #1, 12 μM U0126), p38α/β/β2 (p38i, 15 μM SB203580), or JNK1/2/3 (JNKi, 1.25 μM SP600125) overnight for ~15 h (n ≥ 6–10 and 6–10, resp., mean, s.e.m.), (C-D) intracellular Ca2+ chelator ([Ca2+]i, 5 μM BAPTA-AM) or NCX Ca2+ influx inhibitor (NCXi, 5 μM KB-R7943) for ~3 h (n ≥ 3–10 and 3, resp., mean, s.e.m.), or (E-F) pan Gα subunit (Gαi, ~10 μM BIM-46187), ErbB/HER (EGFRi, 120 nM PD158780), Src (Srci, 350 nM Herbimycin A), or PI3K inhibitor (PI3Ki, 10 μM LY294002; n ≥ 4–8 and 5–6, resp., mean, s.e.m.). EGF (50 ng/mL) was added in (E) as a control for activating HIV-1 gene expression via EGFR/PI3K signaling. Results in graphs (A, C, and E) were confirmed by SDS-PAGE analysis of Gag-GFP expression in Fig 4C and 4D and the inhibitory activity of each kinase inhibitor was confirmed in Figs 4A and 4B and 5A, (E), and/or alongside our previous study. Results are shown relative to DMSO (+) with no kinase inhibitor. Statistical comparisons were performed as illustrated by gray/black-dashed lines (and asterisks) for one of the targets and described in Materials and methods. (TIF) [file ppat.1008307.s018.tif]

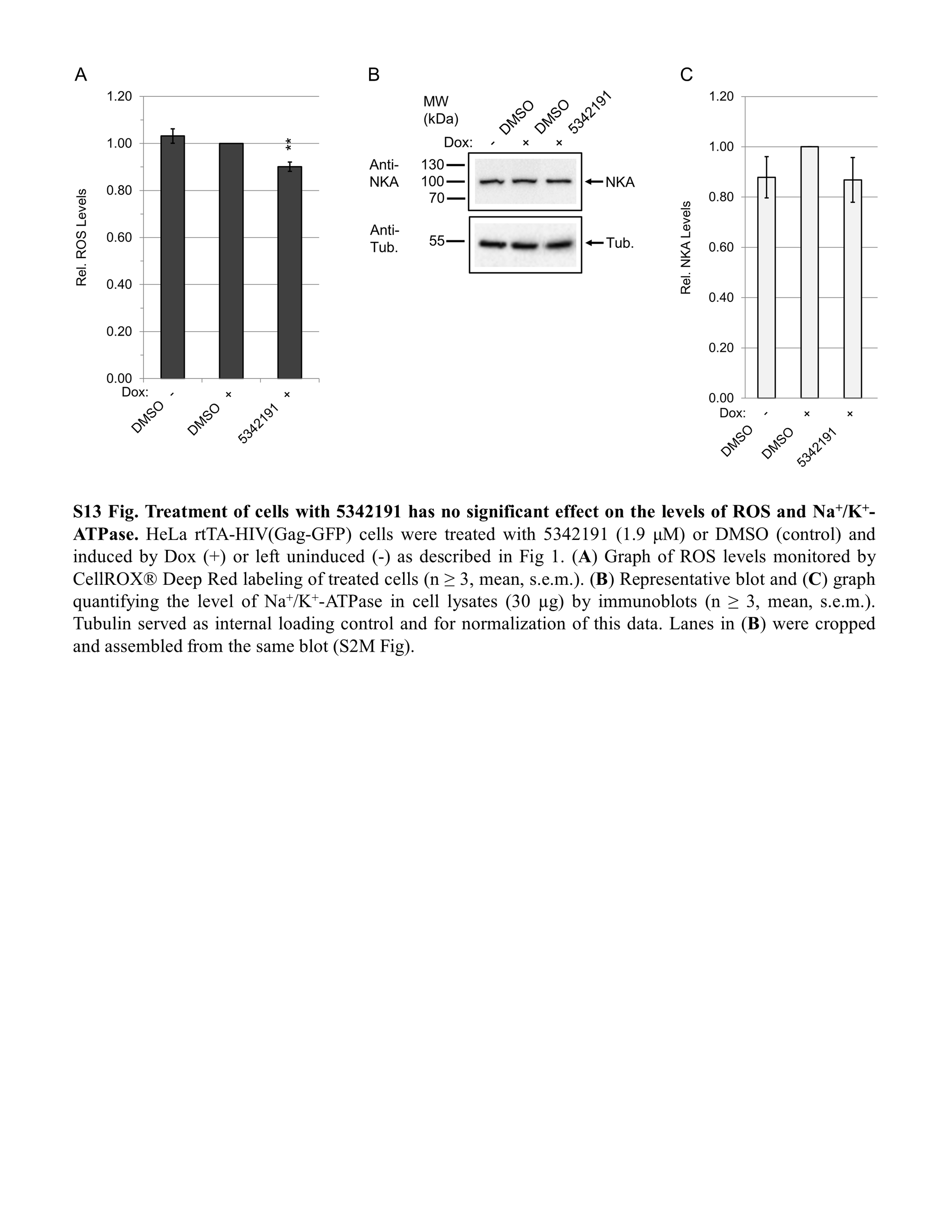

Supplement: S13 Fig — HeLa rtTA-HIV(Gag-GFP) cells were treated with 5342191 (1.9 μM) or DMSO (control) and induced by Dox (+) or left uninduced (-) as described in Fig 1. (A) Graph of ROS levels monitored by CellROX Deep Red labeling of treated cells (n ≥ 3, mean, s.e.m.). (B) Representative blot and (C) graph quantifying the level of Na+/K+-ATPase in cell lysates (30 μg) by immunoblots (n ≥ 3, mean, s.e.m.). Tubulin served as internal loading control and for normalization of this data. Lanes in (B) were cropped and assembled from the same blot (S2M Fig). (TIF) [file ppat.1008307.s019.tif]

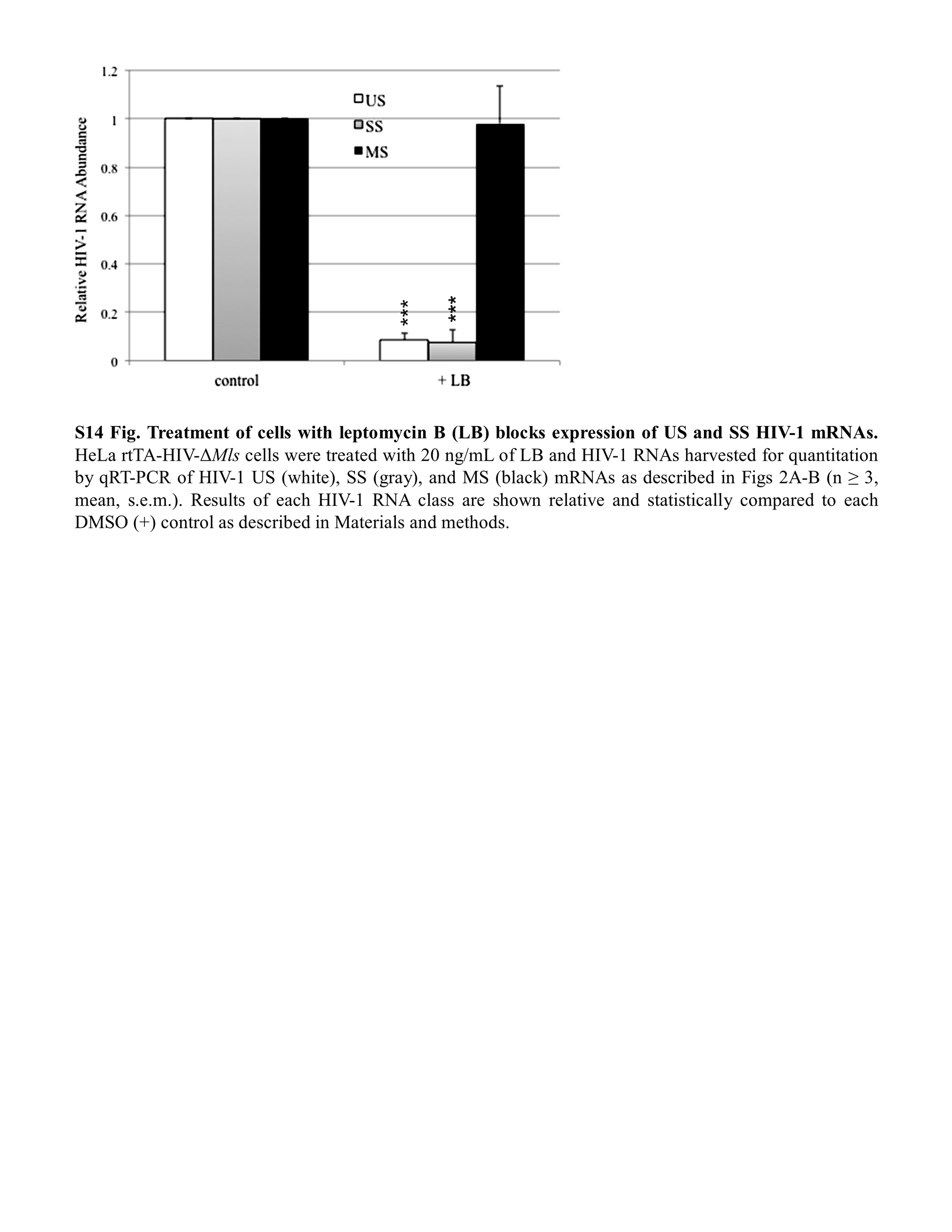

Supplement: S14 Fig — HeLa rtTA-HIV-ΔMls cells were treated with 20 ng/mL of LB and HIV-1 RNAs harvested for quantitation by qRT-PCR of HIV-1 US (white), SS (gray), and MS (black) mRNAs as described in Fig 2A and 2B (n ≥ 3, mean, s.e.m.). Results of each HIV-1 RNA class are shown relative and statistically compared to each DMSO (+) control as described in Materials and methods. (TIF) [file ppat.1008307.s020.tif]

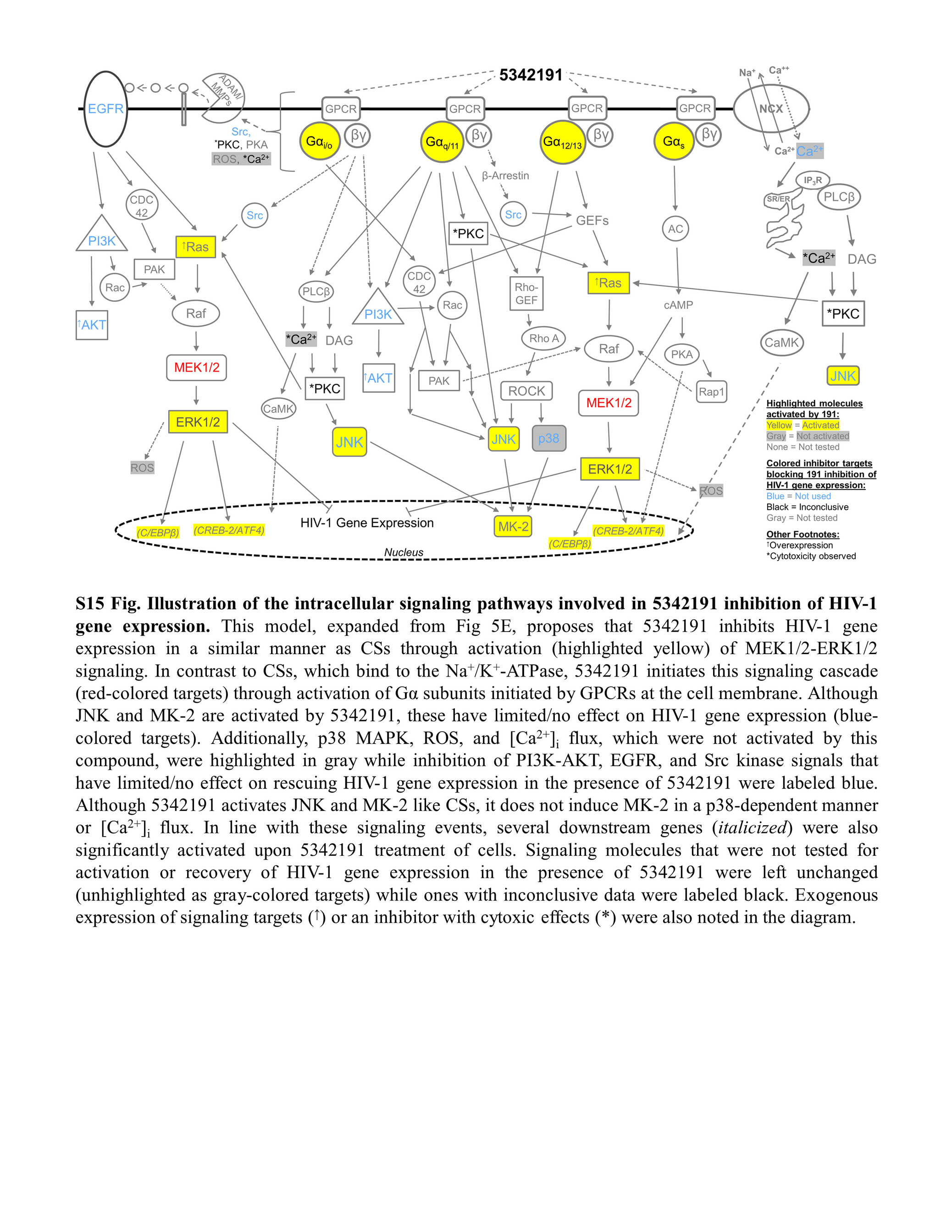

Supplement: S15 Fig — This model, expanded from Fig 5E, proposes that 5342191 inhibits HIV-1 gene expression in a similar manner as CSs through activation (highlighted yellow) of MEK1/2-ERK1/2 signaling. In contrast to CSs, which bind to the Na+/K+-ATPase, 5342191 initiates this signaling cascade (red-colored targets) through activation of Gα subunits initiated by GPCRs at the cell membrane. Although JNK and MK-2 are activated by 5342191, these have limited/no effect on HIV-1 gene expression (blue-colored targets). Additionally, p38 MAPK, ROS, and [Ca2+]i flux, which were not activated by this compound, were highlighted in gray while inhibition of PI3K-AKT, EGFR, and Src kinase signals that have limited/no effect on rescuing HIV-1 gene expression in the presence of 5342191 were labeled blue. Although 5342191 activates JNK and MK-2 like CSs, it does not induce MK-2 in a p38-dependent manner or [Ca2+]i flux. In line with these signaling events, several downstream genes (italicized) were also significantly activated upon 5342191 treatment of cells. Signaling molecules that were not tested for activation or recovery of HIV-1 gene expression in the presence of 5342191 were left unchanged (unhighlighted as gray-colored targets) while ones with inconclusive data were labeled black. Exogenous expression of signaling targets (↑) or an inhibitor with cytoxic effects (*) were also noted in the diagram. (TIF) [file ppat.1008307.s021.tif]
