# Supplementary material for: An activator of G protein-coupled receptor and MEK1/2-ERK1/2 signaling inhibits HIV-1 replication by altering viral RNA processing
Source: PLoS Pathog. 2020 Feb 18;16(2):e1008307. doi: 10.1371/journal.ppat.1008307 (PMC7048317; doi:10.1371/journal.ppat.1008307)
Supplement: Attachment — Submitted filename: ResponseToReviewers.pdf [file ppat.1008307.s022.pdf]

September 26, 2019

Dear Editor(s) of ***PLoS Pathogens***,

We are pleased to resubmit our revised manuscript entitled "**A new activator of G protein-coupled receptor and Raf-MEK1/2-ERK1/2 signaling that inhibits HIV-1 replication by altering viral RNA processing**" (#PPATHOGENS-D-19-00990) as an article for publication. We have addressed the reviewers' comments as detailed in the responses provided below. Please do not hesitate to contact us if there are any other concerns.

Thank you for your time.

Sincerely,

Raymond W. Wong, PhD, *Post-doctoral Research Fellow*
Dept. of Medicine, University of Toronto
67 College St., Rm 453, Toronto, ON, Canada, M5G 2M1
E-mail: rw.wong@mail.utoronto.ca, Tel.: 416-313-4436

PLOS Pathogens: Decision on your submission PPATHOGENS-D-19-00990 - [EMID:02574cd7c6c215c4]

em.ppathogens.0.647ceb.e9a2cb7f@editorialmanager.com
on behalf of PLOS Pathogens <em@editorialmanager.com>
Wed 7/10/2019 5:08 PM
To: Raymond Wong <rw.wong@mail.utoronto.ca>
CC: "Ahalya Balachandran" a.balachandran@mail.utoronto.ca, "Peter K. Cheung" pcheung@cfenet.ubc.ca, "Lulzim Shkreta" lulzim.shkreta@usherbrooke.ca, "Ran Cheng" ran.cheng@mail.utoronto.ca, "Benoit Chabot" benoit.chabot@usherbrooke.ca, "Qun Pan" sandypan2010@gmail.com, "Peter Stoilov" pstoilov@hsc.wvu.edu, "P. Richard Harrigan" richard.harrigan@ubc.ca, "Benjamin J. Blencowe" b.blencowe@utoronto.ca, "Donald R. Branch" don.branch@utoronto.ca, "Alan Cochrane" alan.cochrane@utoronto.ca

Dear Dr. Wong:

Thank you very much for submitting your manuscript "Inhibition of HIV-1 replication by small molecule activation of Raf-MEK1/2-ERK1/2 signaling through G protein-coupled

receptors" (#PPATHOGENS-D-19-00990) for review by PLOS Pathogens. As with all papers submitted to the journal, yours was evaluated by a PLOS Pathogens Associate Editor in consultation with the Editorial Board. Your article was also evaluated by independent reviewers. The reviewers appreciated the attention to an important topic, but they raised substantial concerns about the paper. Based on the reviews, I regret that we will not be able to accept the manuscript for publication in the journal at this time.

As you can see from the comments below, the three reviewers of your manuscript were impressed by the scope of your study and generally believe that the work supports the central premise of your manuscript that that MEK and ERK signaling play a role in modulating gene expression. However, all of the reviewers felt, to some degree, that individual points in your study are not cohesively connected to form a clear picture, and I am inclined to agree with them. Based on this fact, I have made a decision of "Open Rejection" to give you a chance to respond to the this larger issue, as well as the individual issues raised by the reviewers below. I think all of the reviewers raise important concerns that require addressing before your manuscript can be accepted. The singular exception to this would be reviewer 2's concern that the compounds used in this study could cause long term toxicity in patients. While I tend to agree with their concern on this topic, I still feel that the degree to which you have used the agents in the study as tools to reveal novel pathways related to gene expression is significant regardless of the potential utility of the compounds described in your study to act as therapeutic agents, so do not feel that you must perform experiments to counter this concern. However, I think the other concerns raised by this reviewer and the other two are valid and feel that an acceptable revised manuscript would respond effectively to these individual points, with more experimentation where appropriate. As with any Open Rejection, while you are welcome to resubmit this manuscript to PLoS Pathogens, any revision will be reviewed again, by the original reviewers if possible, and as such acceptance cannot be guaranteed.

While the reviewers felt the topic is of interest, they raised substantial concerns that would require major new experiments to address and thus are beyond the scope of a revision. Given the high interest in the topic, however, we would possibly consider a new submission if it were improved to meet the major criticisms of the current manuscript.

The new manuscript would receive a new manuscript reference number but will likely be assigned to the previous editors. It will be their task to determine whether or not to send the manuscript to review and, if so, whether or not to include any new reviewers. We cannot promise peer review or further consideration for publication at that time.

The new submission should make reference to the previous manuscript number in the cover letter and provide a detailed response to the previous criticisms included in the cover letter.

The reviews are copied below, and we hope they may help with any future submissions of this paper to our journal or elsewhere. Please use the link below to see if there are any accompanying reviewer attachments (if no attachments are present, all reviewer comments can be found below).

We are sorry that we cannot be more positive on this occasion, but hope that you appreciate the reasons for this decision and that you will consider PLOS Pathogens for other submissions in the future.

Thank you again for your interest in and support for PLOS Pathogens and open access publishing.

Sincerely,

Thomas J. Hope, Ph.D.
Section Editor
PLOS Pathogens

Thomas Hope
Section Editor
PLOS Pathogens

Kasturi Haldar
Editor-in-Chief
PLOS Pathogens

Grant McFadden
Editor-in-Chief
PLOS Pathogens

****

Thank you for submitting your work to PLoS Pathogens. As you can see from the comments below, the three reviewers of your manuscript were impressed by the scope of your study and generally believe that the work supports the central premise of your manuscript that that MEK and ERK signaling play a role in modulating gene expression.

However, all of the reviewers felt, to some degree, that individual points in your study are not cohesively connected to form a clear picture, and I am inclined to agree with them. Based on this fact, I have made a decision of "Open Rejection" to give you a chance to respond to the this larger issue, as well as the individual issues raised by the reviewers below. I think all of the reviewers raise important concerns that require addressing before your manuscript can be accepted. The singular exception to this would be reviewer 2's concern that the compounds used in this study could cause long term toxicity in patients. While I tend to agree with their concern on this topic, I still feel that the degree to which you have used the agents in the study as tools to reveal novel pathways related to gene expression is significant regardless of the potential utility of the compounds described in your study to act as therapeutic agents, so do not feel that you must perform experiments to counter this concern. However, I think the other concerns raised by this reviewer and the other two are valid and feel that an acceptable revised manuscript would respond effectively to these individual points, with more experimentation where appropriate. As with any Open Rejection, while you are welcome to resubmit this manuscript to PLoS Pathogens, any revision will be reviewed again, by the original reviewers if possible, and as such acceptance cannot be guaranteed.

Reviewer's Responses to Questions

Part I - Summary</br>Please use this section to discuss strengths/weaknesses of study, novelty/significance, general execution and scholarship.

Reviewer #1: Wong et al. describe a small molecule modulator of HIV gene expression, 5342191, and characterize its mode of action.

The first half of the paper describes how 5342191 suppresses HIV gene expression through disruption of viral mRNA alternative splicing, potentially through modulating levels of select SR family splicing factors. The second half of the paper describes how the drug modulates cell signaling in the MEK/ERK pathway and how these effects could operate upstream of HIV expression. Overall, the authors propose that 5342191 is a selective inhibitor of HIV gene expression, and pitch a core biological insight being that MEK and ERK play key regulatory roles in modulating HIV gene expression.

Much of the data is compelling and supports the authors' notion that targeting kinase pathways could be an effective and potentially selective means of suppressing HIV gene expression in vivo. However, the story is complicated with lots of moving parts. It would benefit significantly from additional clarity and some restructuring.

- We understand the reviewer's concern and our entire manuscript has been completely restructured and improved based on all suggestions raised in the reviews. Our total figure count has been reduced by 4. Labels and axes in figures were corrected, simplified, colored, and/or re-aligned as needed to improve presentation of the data (i.e. Figs 1B-C, 1F-G, 1I, 4A-B, and 5A and S11A-D and S14A-F Figs). In simplifying results shown, Figs 4B-F and 4I-J were merged into one sub-figure each (Fig 4A and 4C, resp.), Figs 1B and S3B Fig combined Gag expression and cell viability from two different y-axes into one y-axis (Figs 1B-C), and Fig 2D and S6 Fig were reduced from two lanes into one in representing each treatment on SR proteins (Figs 2I-J and S5 Fig). To reduce confusion, S3B-C Figs were merged with similar contents in figure panels (Figs 1C and 2C, resp.) to eliminate jumping between data files. Data in Fig 4 and 2/3rd of Fig 5 were merged into one Figure (Fig 4) to improve content flow. Figs 3A-C, 4A and 4H, and 5B-C, respectively, were moved to S7A-C Figs, S11A and S11D Figs, and S11E-F Figs to improve emphasis and clarity.

In terms of experiments, a major weakness is that the authors are, ultimately, unable to connect the dots between the two major subplots; kinases and splicing. A congruent model, implied in the abstract, would be that 5243191 is modulating HIV splicing through MEK/ERK signaling, e.g., if N-Ras overexpression or other signals phenocopy 5243191 then it should be demonstrated that this pathway also affects SR protein levels and HIV splicing profiles. Such a link is implied but not yet confirmed.

- We appreciate the suggestions made to improve this manuscript and have addressed this with new data in our revision (Figs 4 and 5). In new experiments, we now show that, while overexpression of Ras is able to reduce Gag expression, the change is not associated with a change in viral RNA accumulation comparable to that seen upon addition of 5342191. Consequently, we now conclude that Ras modulation is not responsible for the effect of 5342191 on HIV-1 RNA accumulation. This conclusion is supported by the observation that inhibition of EGFR or Src signaling also blocks 5342191's effect on Ras but does not alter Gag expression.

Reviewer #2: The major focus of this paper is the drug 5342191, which leads to decreased accumulation of Gag, Env, Tat and Rev. This happens through multiple mechanisms including lower accumulation and nuclear export of unspliced and singly spliced HIV-1 RNAs due to decreased Rev and Tat levels. There were also changes in SR protein levels and phosphorylation, which have been shown by several groups to dysregulate HIV-1 splicing. In addition, 5342191 activated MAP kinase signaling. Surprisingly, the decrease in Tat levels appears to be caused by protein degradation since a proteasome inhibitor increased its expression.

Wong et al make the argument that targeting cellular proteins that regulate HIV-1 gene expression will lead to new therapies. Unfortunately, I am not convinced that there will be selective toxicity for 5342191 in the context of many years of treatment.

While Wong et al report that low numbers of mRNAs had differential splicing events or expression, it is not clear how robust this analysis was or how it should be interpreted. Dysregulation of even a few important mRNAs could have substantial pathological effects in an animal. RNA-seq and proteomics experiments in cancer cell lines makes it hard to extrapolate what the potential effects could be over time and with different environmental challenges that cause specific changes in gene expression. It is also concerning that the drug activates some MAP kinase pathways.

- RNA-Seq and TMT LC-MS/MS data have been updated to report the data as is by the number and percentage of changes observed at a range of cutoffs. The parameters for total RNA/proteins detected and analyzed with confidence are described in our revised Materials and methods and figure legends. The percent of events that each omics analysis represented are also reported in our Results. Although there are limitations in any one of these omics studies, the potential impact of our compound have been addressed at both the transcriptome and proteome levels.
- Despite using a human cervical cancer cell line for our analyses, our evaluation of 5342191 in human $CD4^+$ T cell lines as well as primary T cells (PBMCs) confirm that this compound is well-tolerated by the host. By comparison with other drugs such as CSs, which activate over 5 different signaling pathways (3 MAPKs) and affects ion flux, 5342191 influences only JNK and ERK1/2 in common with these drugs but not p38 or Ca^{2+} flux, potentially offering an explanation of its lower impact on host processes than other HIV-1 RNA processing inhibitors. Furthermore, our preliminary analysis of 5342191 injected into an *in vivo* mouse model of HIV-1 infection demonstrated little difference in survival rates compared to vehicle over a course of 4 weeks with daily IP injection of the compound at a final calculated concentration of 6 µM (N = 3-6, data not shown) . Overall, our data does not indicate any immediate toxic effects of this compound tested *ex vivo* or *in vivo*.

Reviewer #3: The study posits a role for GPCR signaling and assessed several pathways, RNA seq and MS and alternative splicing. The report describes expansive results accompanied by 6Sup Tables and 18 Sup figures- a huge trove of data. The inhibitor drastically reduces HIV structural RNA and protein. It moderately reduced MS mRNA and drastically reduced Tat protein and Rev protein. This reduction is likely sufficient to account for the loss of US and SS mRNA expression.

At the end, it remains unclear if GPCR has much to do with the drastic reduction in the HIV RRE/Rev-dependent mRNAs.

- Although 5342191 does dramatically alter viral US and SS RNA accumulation, the loss of Tat expression cannot account for this effect due to the fact that the provirus used in these assays does not have a functional Tat or TAR. Viral gene expression in the HeLa rtTA-HIV-Δ*Mls* or rtTA-HIV(Gag-GFP) cell lines used are driven by the Tet-ON system. Our demonstration that inhibitors of MEK/ERK or GPCR signaling can block the effect of 5342191 on both viral RNA accumulation and Gag expression does indicate that these signaling pathways are required for the effect of the drug. Our observation that other inducers of the MEK/ERK signaling pathway (e.g. cardiotonic steroids and anisomycin) also cause a similar alteration in HIV-1 RNA accumulation and protein expression further support the importance of this signaling pathway in controlling HIV-1 replication.

Figure 3A evaluates differences in puromycin incorporation by immunoblot between the HIV- (no DOX) and HIV+ and HIV+/Inhibitor. The gel shows a collection of puro-labeled polypeptides as the smear and the majority were collected at the bottom of the gel (dark band). The quantitation shown in Fig 3B (no difference) differs from that by eye (obvious difference), raising the question of what part of the blots was quantified.

- We have updated the figures in our revised manuscript with more representative blots and graph of these results (S7A-C Figs). The bands quantitated are also the ones shown as stated in the revised Materials and methods.

Proteomics results are presented as ion intensities normalized log 2 differences in protein levels and the fold changes are more significant than concluded in the text. The sensitivity of the MS TMT in the presented experiments needs to be made clear.

- TMT LC-MS/MS data was updated to simply report the data by the percentage of changes observed at a wider range of cutoffs in the text (Abstract, Results, and Discussion). The level of detection observed was also briefly mentioned in the Results.

Changes in some mRNAs are observed that could be underlying the proteomics outcome. What is the Raw protein abundance normalized to mRNA abundance? How does this variable change, or not, the differences in protein abundance?

- Although this thought may be an interesting analysis to perform, our comparison of differentially expressed genes from both RNA-Seq and TMT LC-MS/MS at lower cutoff limits (S9E Fig) provided no overlap of targets.

It is known signaling pathways involving Ras and MEK act, with mTOR, to control regulatory translation factors and activate protein synthesis. If 191 inhibit MEK activation

it is expected to diminish protein synthesis. Indeed, Fig 3 shows 191 reduced the de novo protein synthesis, reducing the incorporation of puromycin (in the course of 30 min incubation).

- Our data indicates that 191 activates NOT inhibits MEK signaling. Furthermore, 191 leads to reduced expression of HIV-1 Gag expression (which can be reversed by MEK1/2 inhibitors) with little/no effect on total protein synthesis of the cell. In support of this finding, updated blots of the puromycin labeling of proteins (S7A-B Figs) are provided to better represent the results in an updated graph (S7C Fig).

The Sup Fig 13 B, right lane, and last line of legend: this conjecture may be due to reduced protein synthesis during the 15hr incubation with Sel. Taken together, the data interpretation is equivocal.

- Our data shows that addition of Selumetinib restores Gag-GFP expression in the presence of 191, a response that cannot occur if host translation has been inhibited. Our updated data shows that 5342191 treatment has no significant impact on host protein synthesis (S7 Fig) and, when used in conjunction with Selumetinib/MEKi #2, does not affect total cellular protein abundance as demonstrated by a more accurate representative gel of this result in S11C Fig.

As stated by the authors, the inhibitor result is just like LMB that is inhibiting Rev/RRE-CRM1 dependent nuclear export. Notably, the inhibitor reduced the incorporation of puromycin (30 min exp), indicating protein synthesis was inhibited. Cochrane et al previously showed de novo synthesized Rev is necessary to transactivate HIV RRE-dependent gene expression. Herein the impact of the downregulated Rev compounded with the diminished host protein synthesis is an explanation for the lack of expression of Gag or Gag-GFP.

- Our previous observation (Iacampo *et al*, J Virol. 1996; 70: 8332-8339) demonstrated that continued HIV-1 RNA synthesis (NOT protein synthesis) was required for Rev function. 5342191 has little or no effect on total protein synthesis as measured by the SUnSET assay (see updated blots and quantitation, S7A-C Figs). Despite the reduction in HIV-1 US and SS RNA accumulation seen upon 5342191 addition, there is an increase in viral MS RNA levels indicating that the viral promoter is functioning. The comparison to leptomycin B (LMB) is to underline that the loss of Rev protein upon 5342191 addition (equivalent to Rev inhibition by LMB) could account for the altered viral RNA accumulation alone. That said, we also observe changes in SR protein expression/modification that could result in altered viral RNA processing.

Metabolism marker is reduced by 20% - what is the significance?

- We have included more data points to our previous result (Fig 1E) which initially showed a 20% insignificant drop in cell metabolism/viability with an updated result (Fig 1F) that does not indicate any effects of 5342191 on cell viability.

Part II – Major Issues: Key Experiments Required for Acceptance</br>Please use this section to detail the key new experiments or modifications of existing experiments that should be <u>absolutely</u> required to validate study conclusions.

Generally, there should be no more than 3 such required experiments or major modifications for a "Major Revision" recommendation. If more than 3 experiments are necessary to validate the study conclusions, then you are encouraged to recommend "Reject".

Reviewer #1: 1. Fig2 and Fig3 “-omics”. How selective is selective? The authors emphasize that 5243191 reduces HIV gene expression but with “limited” effects on the cell based on RNA-seq and comparative proteomics (abstract, elsewhere). First off, ”limited” is not a very scientific term- it would be better just to present the data and discuss it as fairly as possible.

- We have reduced the use of this word from the text, replaced it with better word choices, and reported the data in the text as fairly as possible by showing the percent changes observed at two cutoffs.

While I appreciate that the effects may be less than those observed for T cell activation or digoxin treatment (discussion); these analyses are done in HeLa cells and it seems somewhat counter-intuitive to show major effects on SR protein levels (Fig2) and kinase signaling (Fig4) while at the same time arguing that the big data shows little is happening. Moreover, what would be the explanation that HIV splicing is selectively targeted?

- We find that 5342191 specifically reduces expression of HIV-1 Gag (Fig 1) and US and SS RNAs (Figs 2A-C) which, in our new data, can be reversed by specific inhibitors of the activated kinase signals (Fig 4E). In addition, we observed changes in several SR proteins that are known to play a role in regulating HIV-1 RNA processing. Based on our observations, our results indicate that HIV-1 is more sensitive to subtle changes in RNA processing than the host. Supporting this hypothesis, 5342191 causes little/no changes to the host transcriptome, proteome, or cell viability in multiple cell line/types that are not HeLa cells, i.e. 24ST1NLESG and CEM-GXR T cell lines (Figs 1C and 1I, resp.) and primary T cells (PBMCs, Fig 1G). Consequently, it is not that HIV-1 is being selectively targeted

but rather, the complexity of viral RNA processing regulation renders HIV-1 more sensitive to modest changes in host factor activity.

Would this also be true for other genetically-complex retroviruses? If so, why?

- It is too early to speculate whether 5342191 impacts other complex retroviruses (i.e. HIV-2 and SIV) in a similar manner as HIV-1 without further tests. However, the fact that many regulatory sequences and processes that are involved in RNA processing in these retroviruses are conserved makes it likely and worthy of examination outside the context of this manuscript.

Of the genes that are affected is it meaningful? One thing that would help the reader is a much more careful explanation of criteria and what the statistics mean (e.g., why AS and DE were chosen, why a cutoff is a good cutoff, etc.).

- RNA-Seq and TMT LC-MS/MS data have been updated to better describe the parameters used and level of detection obtained in our revised Results, Materials and methods, and figure legends.

Also, the authors should be sure to clearly define cell type, drug concentrations, and time of treatment for all experiments in text and figure legends.

- We agree with this fact and have updated all of our figure legends and text with more detailed information on the cell types, compound/drug concentrations, and times of treatments used for all experiments in the manuscript.

2. Fig 3. The argument that 5243191 drives Tat degradation independent of its effects on splicing is not convincing; MG132 has similar effects on Tat levels in the presence or absence of 5243191. Can the authors resolve what is happening to Tat and Rev.

- The experiment was designed to address the observation that 5342191 addition did not reduce HIV-1 MS RNA levels and yet little or no Tat protein was detected. Consequently, 5342191 could be blocking Tat mRNA translation, enhancing Tat degradation, or a combination of both. Tat is known to be ubiquitinated and targeted to proteasome-dependent degradation (Zhang *et al.* , 2014. *Cell Biosci.* 4(1):61 while degradative ubiquitination of Rev has not yet been reported). Consequently, addition of an inhibitor of the 26S proteasome complex (MG132) to cells would be expected to increase the levels of Tat. The ability of MG132 to significantly rescue Tat expression in the presence of 5342191 when added 8 h prior to cell harvest confirms that Tat is being synthesized (i.e. its mRNA is being translated) in the presence of the compound. In contrast, as shown in our revised figures, we detected no recovery in the expression of Gag or Env in cells treated with MG132 and 5342191 (Figs 3A-B). In the revised manuscript, we now state

that 5342191 is affecting the balance of Tat synthesis and degradation in addition to any effects it might be having on viral RNA processing.

3. Figs 4-6. The MEKi and Gai, and Gaq siRNA rescue results are nice and could be combined into a single figure.

- We support this notion and moved the graphs of MEKi and Gαi into a single figure (Fig 4).

However, the relevance of the N-Ras overexpression expression to 5243191 is not clear. Most importantly, it would be powerful if these the effects of these treatments on HIV splicing were addressed- this would link data from Figs 1-3 to Figs 4-6 in a way that supports a more complete model. Right now one can speculate that N-Ras specifically targets HIV gene expression by modulating splicing but needs to be shown.; i.e., if N-Ras overexpression or other signals phenocopy 5243191 then it should be demonstrated that this pathway also affects SR protein levels and HIV splicing profiles.

- To link the effects of 5342191 treatments on HIV splicing, we determined that the effects observed on HIV-1 Gag and RNA expression are dependent on its activated kinase signals (Fig 4E). In terms of N-Ras overexpression, we have clarified our rationale of these experiments in our revised manuscript. Despite N-Ras overexpression having the same effect as 5342191 on HIV-1 Gag expression and ERK1/2 activation, qRT-PCR of HIV-1 RNAs from these cells (Fig 5D) did not show the same alteration of US and SS viral RNAs or MS viral RNAs. These results indicate that increased Ras activity may not account for the complete response of this compound.

Reviewer #2: 1. In Figure 1F, 5342191 treatment inhibits HIV-1 replication in primary CD4+ T cells, but not nearly as potently as AZT. If this was plotted on a log scale (which would be more appropriate), I don't think the inhibition of replication by 5342191 at 3uM would be impressive. Since 3uM is highest concentration tested in which cell viability was not substantially decreased (Figure 1E), I am not sure if this drug is likely to be potent enough to be an effective antiviral without causing significant side effects.

- We have provided more data on this study and confirmed that 3 μM of 5342191 suppresses HIV-1 growth at a similar level as 3.7 μM of AZT (Fig 1F) with an IC_{50} of ~1.8 μM and demonstrate little/no changes in cell viability at doses tested (Fig 1G). We also find no data to support that this drug causes any significant side effects since our observation on cell viability can be extended to 5 different cell line/types (Fig 1 and S12 Fig) as well as preliminary data (see comments above) in an *in vivo* mouse model of HIV-1 infection.

2. Wong et al show that only 25 splicing events were affected by at least 20% by 5342191, though >300 changes in splicing events were detected if 10% change was used (Figure 2G). However, I am unclear on what the 9,806 exon inclusion/exclusion events that they analyzed represent. Previous studies have shown that there >80,000 splicing events in human cells (e.g. Gerstein et al 2014 Nature 512:445). The authors detected gene expression for 11,406 genes by RNA-seq so it appears that less than one splicing event per gene was detected, which is too low since most human genes have multiple exons. Is this due to low sequencing depth? Is the splicing analysis biased to highly expressed genes and could many more events be changed that did not reach the level of detection in this analysis? Without analyzing more splicing events, I don't see how the authors can be confident that the drug does not cause important changes to cellular RNA splicing.

- We agree with this point and have provided an explanation of the coverage represented by the AS events and mRNA transcripts analyzed by RNA-Seq in the text of our revised manuscript. The primary purpose was not to perform an exhaustive analysis of all possible alterations in host RNA expression but rather to measure whether 5342191 was inducing dramatic alterations in host RNA processing similar to that seen on HIV-1 RNAs. Assays were performed in duplicate and only those events that were consistent among the sample sets are being reported. The 9,806 AS events were analyzed from 18,611 events detected, but, based on our parameters, this data represents ~10% of 100,000 AS events reported for multi-exon human genes. Although this is only a fraction of all possible events, our analysis of the expression of 11,406 genes not only correlate and support the low number of changes to AS but represent >95% of the proteins expressed in all cells (or ~70% of transcript/proteins reported for the human transcriptome/proteome).

3. For the changes in mRNA expression in Figure 2H, the authors found 53 differentially expressed genes with a fold change threshold of 2 and only 1 gene that changed at least 5-fold. However, in the manuscript text, the authors only mention the 5-fold change threshold (page 10), which biases the reader’s interpretation if they are not looking closely at the figure.

- The text has been updated to report the data by the number and percentage of changes observed at both cutoffs of 2-5 fold as suggested.

4. For the host protein analysis, only 18 proteins were shown to change at least 1.5-fold. However, only 5,326 proteins were detected. This seems to bias the analysis to highly expressed proteins. Overall, the authors appear to be trying to convince the reader that the drug is safe, but the host RNA and protein experiments have important limitations.

Only long term treatment of an animal model with the drug will start to show the safety profile of 5342191.

- Our primary goal is to identify novel agents that are able to inhibit HIV-1 replication by a mechanism that is distinct from those of current cART drugs. Application to treatments is likely to require further development but does not diminish the significance of our observations. LC-MS/MS data was updated to report the total proteins analyzed with confidence by parameters described in our revised Materials and methods and figure legends. The level of detection obtained was also briefly mentioned in the Results. Significant changes in host gene expression (at either the RNA or protein level) would be cause for concern but our data indicates that inhibition of HIV-1 gene expression/replication is achievable at doses of 5342191 that have limited or no effects on either of these parameters. We agree that any cell based assay has limitations given that it may not reflect/predict toxicity in animal models. However, the limited effects seen in the cell-based assays has encouraged us to move forward. Preliminary analysis of 5342191 injected into a mouse model of HIV-1 infection demonstrated little difference in survival compared to vehicle over a course of 4 weeks despite daily IP injection of the compound at a calculated final concentration of 6 µM (N = 3-6, data not shown) . Overall, our data does not indicate any overt toxic effects *ex vivo* or *in vivo*.

Reviewer #3: See above concerns to control for the inhibition of host protein synthesis and log2 differences

MG132 may be due to nonspecific effect. More exp would be required to conclude whether or not Tat is less stable or less de novo synthesis is the explanation.

- Although more work will be required to fully elucidate this mechanism, our determination that MG132 addition to cells can partially rescue Tat expression in the presence of 5342191 demonstrates that at least part of the mechanism involved is attributed to activity of the host proteasome. In our revised figures, the response observed is limited to Tat since we did not detect any recovery in expression of Gag or Env in cells treated with MG132 and 5342191 (Figs 3A-B).

Part III – Minor Issues: Editorial and Data Presentation Modifications</br>Please use this section for editorial suggestions as well as relatively minor modifications of existing data that would enhance clarity.

Reviewer #1: 1. Fig1E-H. 3 μM 5243191 reduces but does not inhibit HIV replication (~50% reduction). Care should be taken with the repeated choice of words "inhibit/replication" (title, elsewhere).

- We have taken more care to articulate the effects of this compound on HIV-1 with better word choices and reduced repeated word usage where possible. Figs 1E-H has also been revised with more experimental data (Figs 1F-G) which demonstrates that 5342191 inhibits HIV-1 replication, reaching near an IC_{80}.

Effects are interesting but relative to existing drugs (e.g., AZT in Fig 1F) 5243191 drug is not very potent.

- The main focus of the manuscript is to outline a novel mechanism for the control of HIV-1 replication at the post-transcriptional level, a stage of the virus life cycle not targeted by any of the current FDA-approved drugs. Although 5342191 did not demonstrate nanomolar inhibition of PBMCs infected with HIV-1 *Ba-L*, 5342191 suppressed HIV-1 gene expression at nanomolar potencies in 4 other cell lines infected with different strains of HIV-1 at an average IC_{50} of ~700 nM. Having demonstrated the antiviral activity of 5342191, we are interested in pursuing more extensive medchem optimizations of the compound to increase potency but such studies are outside of our expertise and the scope of this manuscript. Such optimization would be significantly enhanced once we have identified the cellular factor that mediates the response of 5342191 (i.e. the GPCR receptor).

Second, for Fig1H is this really replication (implies measuring GFP levels at a set time point)?

- Understood. We have revised the y-axis of this figure to read Rel. HIV LTR Activity instead of Rel. Level of Viral Replication to reflect the quantitation of GFP fluorescence (Fig 1I).

Experiment and drug concentrations used needs to be better described in in figure and figure legends.

- As suggested, all figures and figure legends were updated with more details of drug/compound/inhibitor concentrations used in each experiment.

Also, how can cell viability be improving for intermediate concentrations?

- Although insignificant, we have noted in the text that CEM-GXR T cells treated for 3 days with 5342191 induced a trending increase in viable cell counts (Fig 1I), which likely resulted from ERK1/2 induction of genes promoting cell proliferation and survival. Furthermore, comparison is to DMSO treated cells infected with

HIV-1, a condition in which cells are affected by the cytopathic effects of the virus.

2. Some of the figure panels should be better designed:
- Figs 1B and S3B. Legend labels are not correct.
    - We can see how these legend labels can be confusing and have, therefore, updated them to reflect one y-axis containing both Rel. Gag Expression and Cell Viability instead of two separate axes (Figs 1B-C). Furthermore, other figure panels have been better designed as mentioned in the first response paragraph provided above.

- Fig2A,E, etc.- Would be easier to follow/interpret if values were normalized to no DMSO control.
    - Unfortunately, all compound treatments are Dox induced (+) to activate HIV-1 gene expression and it would not be correct to use results relative to DMSO treated cells that are uninduced (-). However, normalizing total proteins to the DMSO (-) sample or any other sample would not make any difference in each experiment since results were made relative to DMSO (+).

- Fig1C, etc. consider "vehicle" instead of DMSO (drugs treatments also include DMSO)
    - Although we could use this suggestion instead of DMSO, the same argument could be used to say that all drug/compound treatments include vehicle or solvent. In limiting any confusion, we chose to simply state all control treatments as DMSO or, sometimes, as control.

- Figs 1B, 1E, S3B lack vehicle/negative control
    - The 0 µM used in each of these graphs is the negative control (equivalent to other experiment samples labeled DMSO) and was also noted in our revised figure legends (Figs 1B, 1E, and 1H).

3. Fig 1 and S3B; Related to major comment #1; use of IC50 for Gag expression a bit problematic when for HIV drugs usually refers to infectivity. Try to be clearer here.
    - In clarifying that we are not inhibiting infection of the virus, the first paragraph in the Results of our revised manuscript described IC_{50} as inhibition of HIV-1 gene expression (or replication) and the cell line assayed has an integrated provirus.

4. Page 9; please note, says Fig 1B-D but means Fig 2B-D. "Data not shown" probably not appropriate for this journal.
    - Thank you for this correction. The reference to Figs 1B-D in the text was corrected to Figs 2B-D and the information referring to "data not shown" was removed.

5. Fig2D-E; also big drop in SRSF3, and possible significant drop in total protein in D. E doesn't seem accurate for SRSF1 and SRSFS3 based on blots. Would be better to normalize to no-vehicle control.

- As suggested, better representative blots and graphs of these results are provided in Figs 2I-K of our revised manuscript. However, since 5342191 is solubilized in DMSO solvent, it would be best to normalize results to this vehicle (and not to no vehicle) because of the chance that this may have some effect.

6. S5A-C: the bcl-x splicing assay is interesting but not very clear what it shows, and why overexpressing the SR proteins is relevant to the rest of the paper (not done for HIV). Please explain rationale.

- As inferred by this suggestion, we have removed S5A-C Figs on the *Bcl-x* splicing assay of exogenous expressed SR proteins due to its lack of relevance and confusing nature.

Reviewer #2: 1. The representative blots in Figure 2D do not appear to match the magnitude of the reduction for SRSF1 and SRSF3 in Figure 2E. SRSF1 and SRSF3 appear to be greatly reduced in the blot but not in the graph.

- We agree with this suggestion and have provided better representative blots and graphs of these results in Figs 2I-K.

Reviewer #3: General points: The findings of the study are reiterated over and over in the ms: in the Intro (whole page), the Results section, the Discussion section - both early and late. Redundancy should be eliminated.

- We agree with the reviewer and have reduced many of these redundancies in the revised manuscript.

The title is overly descriptive and should be revised to indicate the impact of the study.

- We appreciate the input on our title and have modified it to better reflect the impact of this study.

The document states rationale to screen for new HIV drugs is the present ART readily generate resistant virus. In this study, the drug is identified, but its capacity to overcome the resistance problem is not addressed, undermining the rationale given for the study.

It is not clear how this drug may be any better than currents drugs in generating resistance. A priori, this drug would also induce resistant HIV quasi-species, adding to the list of compounds usable in HAART regiments. If resistance is not observed, the study would become more significant.

- We agree that this study would be very interesting to explore. However, since our objective was to provide mechanistic insight or proof of concept of a new type of HIV-1 RNA processing inhibitor, we expect to explore better derivatives of this compound as well as validate these molecules in an *in vivo* model of HIV-1 infection prior to exploring extensive 6-month long drug-resistance studies that are outside the primary scope of this study.

PLOS authors have the option to publish the peer review history of their article (what does this mean?). If published, this will include your full peer review and any attached files.

If you choose "no", your identity will remain anonymous but your review may still be made public.

Do you want your identity to be public for this peer review? For information about this choice, including consent withdrawal, please see our Privacy Policy.

Reviewer #1: No

Reviewer #2: No

Reviewer #3: No

In compliance with data protection regulations, you may request that we remove your personal registration details at any time. (Use the following URL: https://www.editorialmanager.com/ppathogens/login.asp?a=r). Please contact the publication office if you have any questions.
