# Supplementary material for: An activator of G protein-coupled receptor and MEK1/2-ERK1/2 signaling inhibits HIV-1 replication by altering viral RNA processing
Source: PLoS Pathog. 2020 Feb 18;16(2):e1008307. doi: 10.1371/journal.ppat.1008307 (PMC7048317; doi:10.1371/journal.ppat.1008307)
Supplement: Attachment — Submitted filename: ResponsetoReview120519Upd121719.docx [file ppat.1008307.s023.docx]

December 9, 2019

**Dear Editors** of ***PLoS Pathogens***,

We are grateful to receive the excellent suggestions made during the review of our revised manuscript. A list of our responses to the review comments are provided below and changes made to the manuscript attached. In addition, we have made the submission requirement corrections requested (12/12/19 and 12/17/19) to the manuscript and this letter. We trust that, with the changes indicated, the revised manuscript will now be acceptable for publication. Please do not hesitate to contact us for any additional details if required.

Thank you for your time.

Sincerely,

**Raymond W. Wong**, PhD, *Post-doctoral Research Fellow*

Dept. of Medicine, University of Toronto

67 College St., Rm 453, Toronto, ON, Canada, M5G 2M1

E-mail: rw.wong@mail.utoronto.ca, Tel.: 416-313-4436

**PLOS Pathogens: Decision on your submission PPATHOGENS-D-19-01870 - [EMID:6aff39901fc9c7c7]**

em.ppathogens.0.6738f1.894b4454@editorialmanager.com

on behalf of PLOS Pathogens <em@editorialmanager.com>

Tue 11/12/2019 3:54 AM

To: Raymond Wong <rw.wong@mail.utoronto.ca>

CC: "Ahalya Balachandran" ahalya.balachandran@gmail.com, "Peter K. Cheung" pcheung@cfenet.ubc.ca, "Ran Cheng" ran.cheng@mail.utoronto.ca, "Qun Pan" sandypan2010@gmail.com, "Peter Stoilov" pstoilov@hsc.wvu.edu, "P. Richard Harrigan" richard.harrigan@ubc.ca, "Benjamin J. Blencowe" b.blencowe@utoronto.ca, "Donald R. Branch" don.branch@utoronto.ca, "Alan Cochrane" alan.cochrane@utoronto.ca

Dear Dr. Wong:

Thank you very much for submitting your manuscript "An activator of G protein-coupled receptor and MEK1/2-ERK1/2 signaling inhibits HIV-1 replication by altering viral RNA processing" (PPATHOGENS-D-19-01870) for review by PLOS Pathogens. Your manuscript was fully evaluated at the editorial level and by independent peer reviewers. The reviewers appreciated the attention to an important topic but identified some aspects of the manuscript that should be improved.

We therefore ask you to modify the manuscript according to the review recommendations before we can consider your manuscript for acceptance. Your revisions should address the specific points made by each reviewer.

In addition, when you are ready to resubmit, please be prepared to provide the following:

(1) A letter containing a detailed list of your responses to the review comments and a description of the changes you have made in the manuscript. Please note while forming your response, if your article is accepted, you may have the opportunity to make the peer review history publicly available. The record will include editor decision letters (with reviews) and your responses to reviewer comments. If eligible, we will contact you to opt in or out.

(2) Two versions of the manuscript: one with either highlights or tracked changes denoting where the text has been changed; the other a clean version (uploaded as the manuscript file).

While revising your submission, please upload your figure files to the Preflight Analysis and Conversion Engine (PACE) digital diagnostic tool, <https://pacev2.apexcovantage.com>. PACE helps ensure that figures meet PLOS requirements. To use PACE, you must first register as a user. Then, login and navigate to the UPLOAD tab, where you will find detailed instructions on how to use the tool. If you encounter any issues or have any questions when using PACE, please email us at [figures@plos.org](mailto:figures@plos.org).

We hope to receive your revised manuscript within 60 days or less. If you anticipate any delay in its return, we ask that you let us know the expected resubmission date by replying to this email.

If you have any questions or concerns while you make these revisions, please let us know.

Sincerely,

Edward M Campbell, PhD
Guest Editor
PLOS Pathogens

Thomas Hope
Section Editor
PLOS Pathogens

Kasturi Haldar
Editor-in-Chief
PLOS Pathogens
orcid.org/0000-0001-5065-158X

Grant McFadden
Editor-in-Chief
PLOS Pathogens
orcid.org/0000-0002-2556-3526

***********************

Dr. Cochrane,
All three reviewers have read your revised manuscript and find it much improved. They do have some minor comments that I feel still require attention. Of these, I would not insist that you address reviewer 3's question about the effect being upstream or downstream of Rev. Although I agree with them that this is an important question, given the scope of the study in its current form I would leave this important mechanistic question for future studies by your group. I would however ask you to address reviewer 1's concerns on the western blot in figure 1D, as it is an important early piece of data providing support for the premise of the study. If you could respond to this comment with an improved western blot and address the other comments of the reviewers, which are largely editorial questions, I would be happy to receive a revised copy of your manuscript.


Reviewer's Responses to Questions

**Part I - Summary**
Please use this section to discuss strengths/weaknesses of study, novelty/significance, general execution and scholarship.

Reviewer #1: The authors have significantly improved the delivery of their study by rewriting of the manuscript text and improving the data presentation. They have answered the critiques appropriately. Minor points remain to be addressed.

The manuscript revision presents a cogent analysis of an inhibitor of HIV mRNA processing. The drug appears to invoke a sensitive response by HIV that diminishes the balanced synthesis of structure and regulatory proteins to attenuate virus replication. The study uses state of the art approaches to measure generalized effect on host mRNAs and specific effect on HIV mRNAs. The text cogently synthesizes a vast dataset into a tenable model.

Reviewer #2: The revised manuscript by Wong et al has addressed my major points. The authors show that 5342191 inhibits HIV-1 replication at concentrations that do not cause detectable cytotoxicity. The mechanisms by which this drug works are complicated, but it appears to act by activating MEK/ERK signaling pathways. This drug may be a useful tool to study how signaling pathways regulate HIV-1 gene expression.

Reviewer #3: This is a resubmission of a manuscript from Wong et al. describing a small molecule inhibitor of HIV gene expression called 5342191 (aka 191). The study is impressive in its breadth- showing the drug to suppress HIV gene expression in several cell types without major effects on cell viability; and featuring large-scale comparative host cell transcriptome and proteome analyses. The major conclusion is that the drug alters HIV alternative splicing through a yet to be resolved mechanism, potentially through modulating the levels or activities of SR family proteins. The authors had previously attributed the splicing effects to 191 triggering MEK/ERK signaling through Galpha and N-Ras. However, based on new data they walk back these claims to the point where they now argue that 191 triggers one of potentially multiple complex signaling pathways that link GCPRs and HIV-1 splicing program. Overall the authors have done a good job of responding to reviewers' comments and re-working a lot of interpretations. There are remaining mechanistic gaps and a persisting, unnecessary desire to argue that the drugs don't affect cell physiological; but overall the paper is improved.

**Part II – Major Issues: Key Experiments Required for Acceptance**
Please use this section to detail the key new experiments or modifications of existing experiments that should be absolutely required to validate study conclusions.

Generally, there should be no more than 3 such required experiments or major modifications for a "Major Revision" recommendation. If more than 3 experiments are necessary to validate the study conclusions, then you are encouraged to recommend "Reject".

Reviewer #1: There is need to make equivalent loading control in figure 1D. Lane 3 underloaded compared to lane 2, making it ineffective to compare the HIV protein signals between the -/+ drug treatment.

- We have replaced Figure 1D with a properly loaded immunoblot to allow better comparison between the expression of HIV-1 Gag and Env in cells treated with/without compound 5342191.

Reviewer #2: None

Reviewer #3: Figs 2 and 3. It’s still strange that Tat and Rev expression is abolished by 191 if the host proteome is largely intact (and seems unlikely its degradation…). It still seems to be a key question as to what is happening to Rev and Tat despite high levels of MS transcripts, and if loss of Rev really underpins the overall defect. This is handled better in the discussion than before, but can the authors resolve if 191 is acting upstream or downstream of Rev?

- This is an interesting point. The experiments using MG132 provide some evidence that Tat is at least partially affected by degradation but do not rule out the possibility of the compound affecting translation of MS RNAs. Further investigation will be necessary to determine whether Rev is affected by 5342191 in a similar manner as Tat and if it acts upstream or downstream of this factor. These studies have been hampered by the poor quality of anti-Rev antibodies that are commercially available. Our data demonstrates that 5342191 activates intracellular signals that reduce US and SS RNAs by 60% and, through our analysis of US HIV-1 RNA localization, supports that loss of Rev expression provides one explanation for the nuclear sequestration of the remaining 40% of US RNAs that could, otherwise, be exported and expressed in the cytoplasm.

**Part III – Minor Issues: Editorial and Data Presentation Modifications**
Please use this section for editorial suggestions as well as relatively minor modifications of existing data that would enhance clarity.

Reviewer #1: P9 typo .........with the exception of SRSF1 (~2-fold decrease)… with the exception of SRSF1 (~50% NOT 2-fold decrease), .........and SRSF3 (~30% decrease),

- For clarity, we have modified the sentence to “Comparison of 5342191 versus DMSO-treated cells (Figs 2I-K and S5 Fig) revealed no significant change in the abundance of most SR proteins examined with the exception of SRSF1 (~2-fold decrease to 50%), SRSF4 (2.1-fold increase to 210%), and SRSF3 (~30% decrease to 70%), all of which have been shown to affect HIV-1 RNA processing and expression [27,48–55].” We also modified a similar sentence in the Discussion to “Consistent with this conclusion, treatment of cells with 5342191 altered the abundance/modification of a subset of SR proteins known to regulate HIV-1 RNA processing (Fig 2K) [27,42,43,48,50,54,55,68]: increasing SRSF4 levels (abundance and modification) by 2.1 fold (to 210%) and decreasing SRSF1 and SRSF3 abundance by 2 fold and 30% (to 50% and 70%, resp.) relative to controls, without altering SRSF 6, 7, 9, and Tra2α levels.”

P30 top, first word correction to: use
Instead of ….used replaces “kinase inhibitor”

- This sentence needed to be corrected to “Alternatively, MG132 replaces “kinase inhibitor” described above.”

Figure 1D/ Gag WB: Gapdh exposure + DMSO (lane 2) is >>>than Gapdh +DMSO plus 5312191 (lane 3). The lanes require equivalent loading.

- We addressed this issue with an alternative set of immunoblots for Fig 1D.

Need to address the following:
From PMID: 30118183…..Glycogen Synthase Kinase-3 (GSK-3) is a highly conserved negative regulator of receptor tyrosine kinase, cytokine, and Wnt signaling pathways. GSK-3 regulates alternative splicing in response to T-cell receptor activation, and recent phosphoproteomic studies have revealed that multiple splicing factors and regulators of RNA biosynthesis are phosphorylated in a GSK-3 dependent manner. The inhibition of GSK-3 alters the splicing of hundreds of mRNAs, indicating a broad role for GSK-3 in the regulation of RNA processing. GSK-3 regulated phosphoproteins include SF3B1, SRSF2, PSF.
1. Since GSK-3 is a critical regulator of mRNA processing, how do the kinase inhibitors used in this study affect GSK3 substrates?

- Given the role of GSK3 on alternative splicing of RNAs, this is an interesting kinase to explore. Analysis of our proteomics data shows that 5342191 causes less than a +1% and +7% change in expression of GSK3A and GSK3B, respectively (S4 Table). Whether this compound regulates GSK3 activity (i.e. post-translational modification) and its substrates would be interesting to explore. However, this kinase was not among any of the phosphorylated proteins detected by TMT LC-MS/MS (data not reported).

2. Since PSF is known to regulate balanced splicing of HIV-1 (PMID: 29846681), what is the impact of this 5312191on PSF activity and how much is the contribution to the reduced replication?

- Our data shows that 5342191 causes only a +3% change in expression of SFPQ/PSF. Whether this compound impacts PSF activity and HIV-1 replication is unknown and, unfortunately, was not among the phospho proteins detected by our proteomics analysis (data not shown).

I suggest the title is further improved to focus on the primary aim: Targeting HIV mRNA processing efficiency is a valid strategy for antiviral drug development.

- We appreciate this broader title but we chose to retain the current title.

Note: An entire page of the Introduction text is summarizing the study, which seems excessive and unwarranted and redundant.

Reviewer #2: In Figure 4E, is the color code correct? The SS and MS colors appear reversed on the figure relative to the legend.

- We recognize the error and have corrected the colors in this graph to match the legend.

Reviewer #3: 1. General- still some uses of diminutives (“little/no”, “minor”, “minimal”, etc.) in referring to changes to host processes in response to 191 (P3, P5, P8, P10, P17); again, considering big effects on MEK/ERK, SR proteins, cell proliferation, etc., and discernible changes in big data, seems inappropriate and unnecessary to use these terms.

- We have corrected the choice of these words as suggested by changing the following: little/no to no significant or no substantial, minor to less than a 0.7%, very small changes to less than 0.7%, very minimal alterations to less than 0.7% of alterations, and minimal to only 0.02-0.34% change.

2. In general, many experimental details useful to the reader (nature of assay, drug concentration, timing, etc.) are still lacking throughout the results section.

- As suggested, we have added more experimental details to appropriate parts of the Results, Discussion, Material and methods, and Figure legends of the manuscript.

3. Abstract- S3- mean “total cell protein synthesis”; S4- still seems uncertain that US lack of transport to cytoplasm is due to loss of Rev. S5- no evidence that Tat levels are due to degradation effects.

- We have corrected S3 to total protein synthesis of the cell. Since our FISH results confirm a lack of transport (of US RNAs, we feel that the observed loss of Rev is the simplest explanation of these observations. In terms of Tat, we find it reasonable to describe that 5342191 affects either the degradation, translation, or both by “suggesting that this compound alters the synthesis/degradation of this key viral factor.” Since addition of MG132 is able to restore some Tat expression in the presence of 5342191, it indicates that synthesis of the protein is occurring.

4. Figs. 1B and 1C. Why are the X-axes not equivalent for these experiments?

- The X-axes between Figs 1B and 1C are not equivalent because these are two independent studies of the compound on two different HIV-1 infected cell lines, rtTA-HIV-ΔMls HeLa and 24ST1NLESG T cells. Additionally, the dose response of the compound on Gag and cell viability that were run in parallel are not equivalent for the former, especially at lower concentrations, because a different dilution series and cell culture format were used in these assays. We also recolored the dose-response curves on the impact of 5342191 on HIV-1 Gag expression from black to red to improve visual comparisons.

5. Fig. 1G. The authors suggest an IC50 for HIV gene expression of 750 nM based on Figs. 1B and 1C but saw no effect at this concentration over six days in their replication experiment. Please resolve.

- Some of the differences observed could be attributed to the nature of the cell signaling induced within each of the cell types utilized. Both the HeLa and SUPT1 cell lines used in Figs 1B and C are transformed cell lines that likely have alterations in cell signaling that confer the transformed phenotype. Alternatively, it might be due to differences in expression of the specific GPCR affected by 5342191 or clearance of the compound over the course of the assay. We have provided more details on the nature of this experiment in the Results and Material and methods sections to note that this compound was only partially replenished once on day 4 in the experiments using primary cells and provide a more detailed explanation of why we observed a difference in the dose-response curves provided.

6. Fig. 1H-I. Would be best if consistent and show concentrations of drug on X-axes. Also, if the effects are due to splicing then the assay is not measuring LTR activity. Please correct.

- We have corrected this graph to show the range in concentrations of compound used to treat cells in this study. Since this experiment uses a CEM-GXR T-cell line containing an integrated LTR-GFP reporter requiring Tat for activation (by various strains of post-integrated HIV), this assay is a an indicator of HIV-1 replication within the culture.

7. Fig. 2B, C, etc. The authors argue in their response that RNA levels should be normalized to the Dox or PMA conditions but seems more useful to the reader to normalize to the negative or baseline.

- The Dox or PMA –‘ve samples should not be used as baseline because these are uninduced cells containing a Tet-ON or naturally activatable HIV-1 provirus that should, theoretically, not express any viral RNAs. Dox or PMA –‘ve samples are best described as background and, if anything, these values could be subtracted from all samples assayed rather than be a denominator of these results.

8. Fig. 5D. Problem here with the labels? Should be US SS MS?

- We appreciate the attention to this overlooked detail and have fixed this graph to correctly match the labels.

9. Fig S2. While it is good to see the original blots included, they are not very interpretable with only cherry-picked labels.

- We are simply adhering to universal guidelines of ethically reporting our results and chose not to add extraneous labels because this would lead to unnecessary complexity and confusion for our readers.

10. Fig S5 seems to show a major 191-driven loss to SRSF2, SRSF7, SRSF9, and Tra2beta expression that is not reflected in Fig 2K. Please explain/resolve.

- We have made a note to explain/resolve any inaccurate interpretation of this data from its protein load in the text of the figure legend.

PLOS authors have the option to publish the peer review history of their article ([what does this mean?](https://journals.plos.org/plospathogens/s/editorial-and-peer-review-process#loc-peer-review-history)). If published, this will include your full peer review and any attached files.

If you choose “no”, your identity will remain anonymous but your review may still be made public.

**Do you want your identity to be public for this peer review?** For information about this choice, including consent withdrawal, please see our [Privacy Policy](https://www.plos.org/privacy-policy).

Reviewer #1: No

Reviewer #2: No

Reviewer #3: No

*In compliance with data protection regulations, you may request that we remove your personal registration details at any time.* [*(Remove my information/details)*](https://www.editorialmanager.com/ppathogens/login.asp?a=r)*. Please contact the publication office if you have any questions.*

As requested under your submission requirements (12/12/19), we have added more detail under our Ethics Statement naming the institution’s review board approving our procedures. We have removed the redundant Author Contributions and Funding information from the Acknowledgements section since this was already added during submission. And, we have removed text that described our class of compounds as “unpublished” from the Results section as well as replaced any “data not shown” with the appropriate described data (S9F) in the manuscript.
