# Supplementary figures and images for: Correction: An activator of G protein-coupled receptor and MEK1/2-ERK1/2 signaling inhibits HIV-1 replication by altering viral RNA processing
Source: PLoS Pathog. 2024 Apr 9;20(4):e1012155. doi: 10.1371/journal.ppat.1012155 (PMC11003610; doi:10.1371/journal.ppat.1012155)

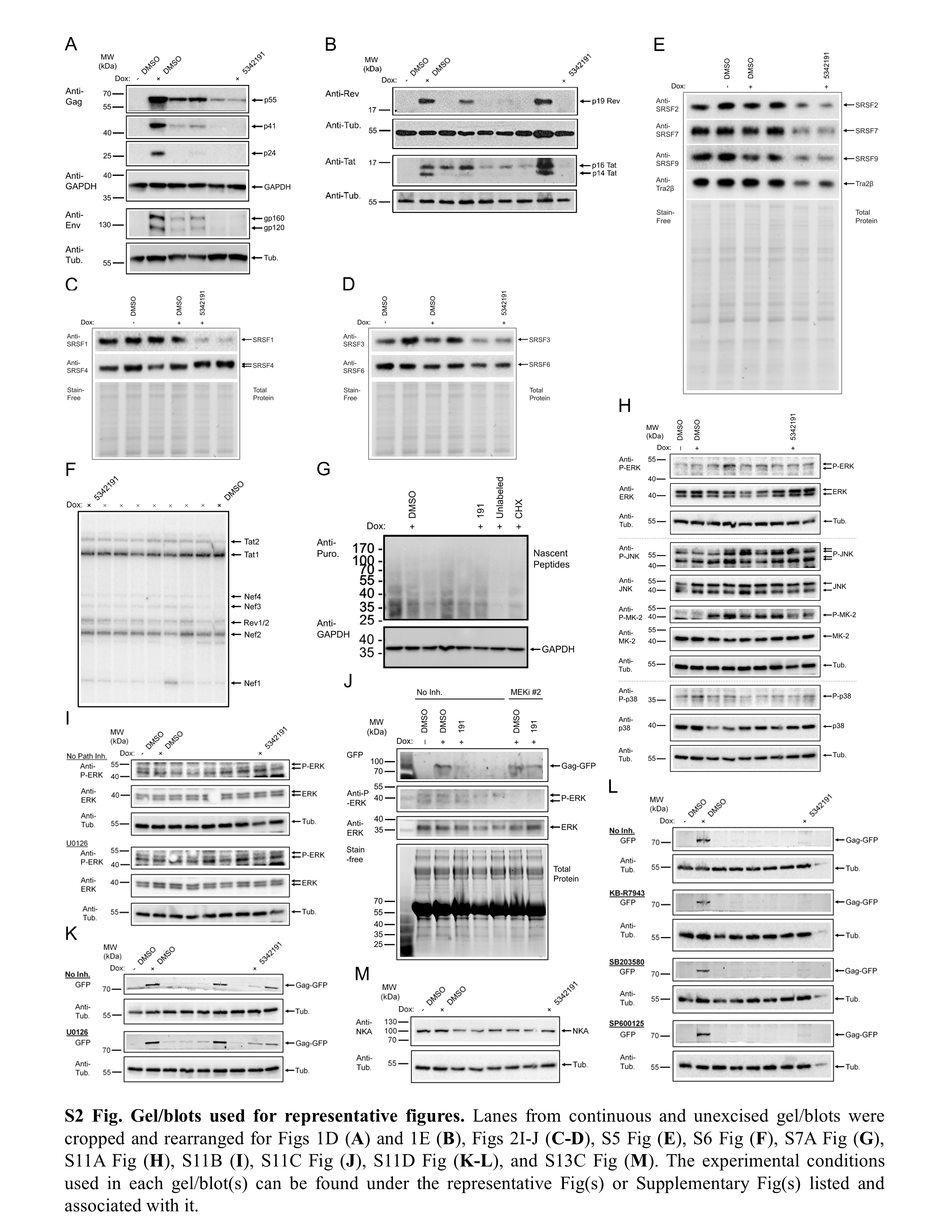

Supplement: S2 Fig — Lanes from continuous and unexcised gel/blots were cropped and rearranged for Fig 1D (A) and 1E (B), Fig 2I and 2J (C-D), S5 Fig (E), S6 Fig (F), S7A Fig (G), S11A Fig (H), S11B (I), S11C Fig (J), S11D Fig (K-L), and S13C Fig (M). The experimental conditions used in each gel/blot(s) can be found under the representative Fig(s) or Supporting Fig(s) listed and associated with it. (TIF) [file ppat.1012155.s001.tif]
